# Exploration of the Electronic and Catalytic Properties of [Co_5_MS_8_(PEt_3_)_5_]^1+^ Nanoclusters: A Computational Study

**DOI:** 10.3390/nano16100587

**Published:** 2026-05-12

**Authors:** Shana Havenridge, Audrey Grace Miller, Cong Liu

**Affiliations:** 1 Chemical Sciences and Engineering Division, Argonne National Laboratory, Lemont, IL 60439, USA; 2 Illinois Mathematics and Science Academy, Aurora, IL 60506, USA

**Keywords:** nanocluster, catalysis, electronic structure

## Abstract

Recent studies have demonstrated the relative stability of undercoordinated hexanuclear cobalt sulfide nanoclusters (NCs) with different charge states. Considering that these small metal NCs have atomically precise structures and high reactivity due to the open shell of the transition metals, and provide selectivity toward ligand loss, they are a vital model for catalysis. In this paper, the electronic structures of these NCs are investigated. These NCs are then used as the reference state to analyze the catalytic properties with respect to hydrogen evolution reaction (HER) and CO_2_ reduction (CO_2_R). Further, to understand the effect of heteroatom incorporation, the geometry and reactivity of ten different metal dopants are analyzed. This work shows that the type of metal incorporation greatly affects the electronic structure and formation energies for ligand binding and catalysis. Particularly, the d-orbital occupancy in the cobalt atoms remains largely unchanged, while the heteroatom greatly influences the reactivity of the undercoordinated NCs. Most notably, this work highlights that transition metals in [Co_5_MS_8_(PEt_3_)_5_]^1+^ NCs would competitively prefer electrochemical adsorption of H over COOH, while the main group metals prefer COOH adsorption.

## 1. Introduction

Through extensive efforts, small metal nanoclusters (NCs) have proven to be efficient catalysts due to their definitive structure and selectivity. Transition metal NCs, specifically, are an ideal model for catalyst design due to the metal’s higher reactivity [1]. Ni_6_(SC_2_H_4_Ph)_12_, for example, has shown enhanced hydrogen production [2], while efficient photocatalytic CO_2_ reduction (CO_2_R) was performed with [Cu_6_(HPymSH)_2_(PymSH)_4_]^2+^ and [Cu_6_(PymSH)_2_(PymSH)_4_] NCs [3]. Considering the atomically precise nature of these NCs, the properties and selectivity can be tuned by changing the size, shape and ligand structure, allowing for better control for a desired product [4].

While transition metal NCs have shown to have tunable, atomically precise control in synthesis, the reactivity is enhanced when the NCs are undercoordinated, leaving an unencumbered area for small molecule adsorption. This allows for better catalytic pathways on the cluster through the undercoordinated metal site and additionally provides less steric hinderance for small molecule adsorption [5]. The cleanest example of this can be seen with reversible ligand dissociation in metal carbonyl NCs in which the CO ligand may be removed thermally or photochemically, leading to undercoordinated metal centers for catalytic applications [6]. Unfortunately, these NCs show strong electronic coupling to CO that often leads to aggregation or cluster decomposition after the ligand is lost [7]. Noble metal NCs have been explored to create more stable, persistent undercoordinated metal sites that more efficiently operate under catalytic conditions without immediate collapse [8,9]. Unfortunately, with more stability comes less reactivity. To remedy the gap between reactivity and stability, atomically precise transition metal NCs have gained interest, specifically those in which the cluster remains stable after ligand dissociation. This paper serves to dive deeper into these NC systems to calculate their structure and thermodynamic properties for the potential catalytic applications.

In metal chalcogenide NCs, specifically of type Co_6_S_8_L_6_ (L = ligand), the ligands can be systematically removed in the cationic, neutral and anionic charge states with blue light or heat [10,11]. These undercoordinated NCs can then be deposited onto self-assembled monolayer (SAM) surfaces, giving well-defined NCs that stay intact on the surface [12]. Given the tunability and precision of these NCs at the atomically precise scale, there is an incredible opportunity for catalysis in these NCs, which has not yet been explored in the literature. It is important to note that single-site catalysis has been explored using these NCs as a support, with an additional metal in the ligand framework acting as the catalytic site to promote charge transfer [13]. This work explores these NCs as single site catalysts.

Studies have further shown that the multi-metallic nature of these NCs can be tuned by incorporating other metals into the core. [Co_5_FeS_8_(PEt_3_)_5_]^+^ NCs, for example, remains as an unreactive monomer on SAM surfaces, while [Co_5_MS_8_(PEt_3_)_6_]^+^ (M = Co, Mn, Ni) undergoes selective dimerization [14]. The effect of the heteroatom has been discussed in these metals, where significant differences in ligand binding energies were obtained [15]. This paper discusses different heteroatoms and analyzes how the electronic density between different metal atoms changes the structure and small molecule adsorption of the catalysts.

In our previous study, we analyzed the collision cross-section (CCS) of [Co_6−x_Fe_x_S_8_(PEt_3_)_6_]^+^ (x = 0, 1, 3, 6) NCs compared to experimental structures characterized by electrospray ionization mass spectrometry (ESI-MS) [11]. Our calculations with density functional theory (DFT) matched within 3% of the experimental values. Recently, we also looked at the relative stability of each [Co_6−x_Fe_x_S_8_(PEt_3_)_6_]^+^ NC towards fragmentation compared to energy-resolved collision-induced dissociation (CID) experiments [16]. The phenomenal similarity between the computational results and experiments reassures confidence for predictive design of these NCs using computational methods, and based on the above observations, we hypothesize that the change in electronic structure upon metal incorporation could in turn lead to change of catalytic properties.

**Figure 1:**
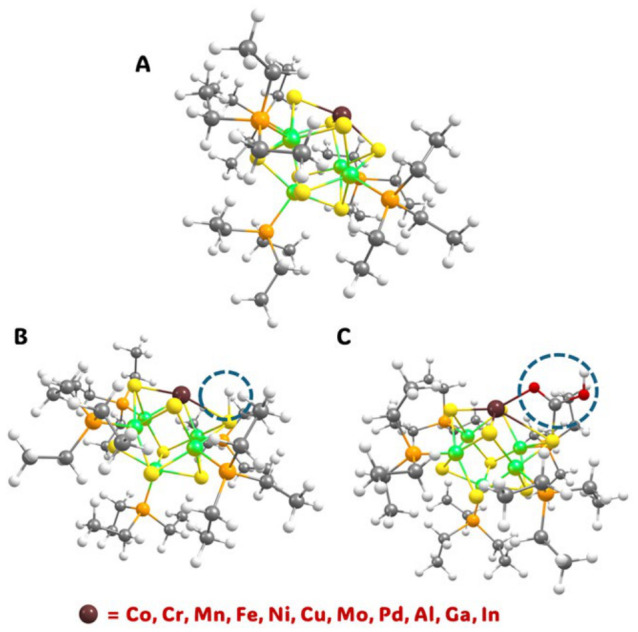
Picture of [Co_5_MS_8_(PEt_3_)_5_]^1+/2+^ NCs (**A**) undercoordinated (L5) (**B**) with hydrogen adsorbed (H*, circled) on the sulfur site and (**C**) with COOH adsorbed on the sulfur site (*COOH, circled).

This work investigates these NCs one step further by analyzing a set of ten different heteroatom dopants into the [Co_5_MS_8_(PEt_3_)_6_]^+^ framework, specifically M = Cr, Mn, Fe, Ni, Cu, Mo, Pd, Al, Ga, In, and compares the results to the parent Co_6_ NC. This work analyzes different isomers and spin multiplicities of each NC to obtain the best model for electronic structure. An in-depth NBO analysis of each NC is then completed to explain the d-orbital occupancy and discuss how this change leads to different to ligand binding energies. Lastly, as shown pictorially through Figure 1, this work analyzes the lowest energy binding site and reaction energies for small molecule adsorption by using the CHE model to evaluate the thermodynamic energies of the hydrogen evolution reaction (HER) and CO_2_R to CO. To do this, we conduct a similar study to Au_24_M NCs completed by Rybacki, et al. [16] by addressing the competitive reaction scheme between HER and CO_2_R, we similarly provide a linear trend to analyze different dopant metals with respect to catalysis. Considering the excellent match to experimental data in our previous studies, this study will aid further experiments, guiding catalytic reactions in lab and providing a vital foundation into heteroatom incorporation with these NCs.

## 2. Methods

### Computational Details

All geometry optimizations were completed with density functional theory (DFT) in ORCA 5.0.3 [17], at the PBE0-D3/def2TZVP level of theory. PBE0 is a one parameter hybrid functional with 25% exact Hartree-Fock exchange [18], ‘-D3′ incorporate dispersion effects according to the Grimme3 model [19], and def2TZVP is an all-electron valence triple-ζ basis set with polarization functions [20,21]. The RIJ(COSX) approximation was used [22], and all calculations were completed in the gas phase without symmetry constraints. As these clusters have an open electronic shell, several spin multiplicities were optimized, and the lowest energy was deemed most stable. To verify the spin multiplicities, spin-polarized geometry optimizations were completed in Vienna ab initio Simulation Package (VASP), version 5.4.4, with the PBE functional to obtain the magnetic moment of each NC [23,24]. VASP calculations were completed with the projector augmented wave (PAW) method to represent the ionic cores [25], with a cutoff energy of 400 eV and gamma k-points. Natural bond orbital analysis (NBO) [26,27] and frequency calculations were completed on the lowest energy spin state for each NC using PBE0-D3/CEP-31G in Gaussian 16 [28], where CEP-31G is an effective core potential double-ζ basis set [29,30]. The zero-point energy and Gibb’s free energy corrections from the frequency calculations were added to the absolute energy from ORCA to calculate ΔE_ZPE_ and ΔG_298.15_ values (def2TZVP(opt)/CEP-31G(freq)). This is demonstrated in the supporting information (Table S17). Partial oxidation states were calculated using the concepts and perl script provided in the work of Webster et al. [31,32], and Patel et al. [33]. Using NBO calculations, the natural atomic orbitals (NAO) occupation matrix was diagonalized, resulting in eigenvalues (between 0 and 1) that represent the d-orbital occupancy. These values are then used to establish a partial oxidation state using different threshold values [31]. As NBO calculations decompose electronic density into lone pairs, bonding and antibonding orbitals, orbitals can have fractional occupancies, especially in these NCs that have superatomic, or delocalized density across the core. The threshold value decides the cutoff occupation number. While the threshold is unfortunately not well defined in these clusters for POS due to the uncertainty of the electron withdrawing nature from the ligands. We used 65% bonding character in Tables S18 and S19. Considering this can still have ligand character, we omitted full analysis with POS and continued analysis with d-orbital occupancy.

## 3. Results and Discussion

Initially, ten different metals were chosen to incorporate into the [Co_5_MS_8_(PEt_3_)_6_]^+^ framework. Most of the dopant metals are located in 3d and 4d periods, including NCs that have been experimentally verified such as M = Mn, Ni, and Fe [14]. Additionally, since aluminum clusters were one of the first studies to verify the superatom concept [34], which provides critical foundation to the superatomic nature shown in these NCs [35,36], main group metals M = Al, Ga, and In were additionally studied. One Co atom was replaced by the dopant metal, and the lowest three spin multiplicities were optimized with DFT; the lowest in energy was deemed most stable. Specific details are described in the Section 2. Notably, the level of theory comes from our large benchmark between DFT functionals on 17 different 3d transition metal nanoclusters [37]. The spin state for each fully ligated NC can be seen in Table S1. Chromium is unique in that it is the only metal that has spin multiplicities close in energy (<0.20 eV). Specifically, the triplet and quintet spin state are within 2 × 10^−3^ eV (Table S2). The focus of this work is one metal incorporation; however, some metals can sequentially replace up to all six Co atoms in this NC. To obtain additional trends, calculations were completed with up to three metal incorporations. The relative energies between spin states and isomers for multiple dopant atoms can be seen in Tables S2–S5. Furthermore, since the predicted oxidation state in the parent NC, [Co_6_S_8_(PEt_3_)_6_]^+^ is 2 for all Co atoms [35,36,38], the study was repeated with the oxidized NC, [Co_5_MS_8_(PEt_3_)_6_]^2+^ for M = Al, Ga, In and Cr, since the metal prefers a +3 oxidation state (Table S6). To obtain the magnetic moment, all the NCs were additionally optimized in VASP with a plane wave basis set as shown in Table S7. The good agreement between the magnetic moments from the plane wave basis set and spin multiplicities obtained from the all-electron basis set provide confidence that the model is not over localizing the electronic density in these systems. Analyzing the SOMO–LUMO gap of the NCs (Table S8), it is apparent that the Ni and Pd doped systems have a 0.13 eV and 0.27 eV larger gap than that of the parent cluster, hinting that the metal incorporated NC is slightly more stable in these systems. This is because Ni and Pd are diamagnetic (all electrons are paired), while every other NC is paramagnetic. Compared to the parent NC in the neutral charge state, all NCs are less stable. There is no common trend across the periods, but the gap gets smaller as the main group metals get bigger as one moves through the group. It is notable that, as more metals are incorporated, the NC has lower SOMO–LUMO gaps in the cases of Cr, Mn, Fe, Ni, Pd and Al dopants, and the parent Co_6_ NC. Additionally, Mo approaches the gap of the parent NC with three Co atoms replaced. Like mono-doped Ni and Pd, this is because [Co_3_Mo_3_S_8_(PEt_3_)_6_]^1+^ is diamagnetic. With respect to geometry (Table S9), all 3d metals have a larger average Co–Co bond distance and smaller Co–P bond distance except for Cr, that has the opposite trend. With the main group and 4d metals, as the metal atom gets larger, the average Co–Co and Co–M bond distances get larger, while the Co–P bond distances get smaller. There is no common trend with M–P bond distances; however, it is notable that the diamagnetic NCs (M = Ni, Pd) have the two smallest M–P bond distances with a value of 2.172 Å and 2.266 Å, respectively.

In our previous study, the fragmentation pathway of these NCs was examined. This study showed with energy-resolved collision-induced dissociation (CID) experiments that it is more favorable for these NCs to lose a ligand compared to a sulfur atom [15]. Hence, the undercoordinated NCs were analyzed with DFT by removing a neutral PEt_3_ ligand, e.g., [Co_5_MS_8_(PEt_3_)_5_]^1+/2+^. Different spin multiplicities and configurations were calculated, where the lowest energy was deemed most stable (Table S10). For the undercoordinated NCs, the ligand can be removed from any of the six metal systems. Initially, only a few symmetric isomers were completed for each metal. To be specific, the initial starting structures contained three possible isomers as seen in Figure S1: the ligand removed from metal atom, the ligand removed from Co across from the metal (Co-trans), and the ligand removed from Co next to metal (Co-cis). These initial geometries were used as symmetrically equivalent positions should have the same absolute energy. This is not the case in this family of NCs, as the isomers showed relative energy values that were not degenerated. Considering that the flexibility in the ethyl ligands can reorient the NC to a more stable structure, Jahn-Teller distortion is possible in these NCs, leading to differences in energy that are unexpected from isomers with the same symmetry [15]. Each ligand was therefore removed from each metal in the NCs with <0.15 eV relative energy of the initial configurations. The lowest-energy spin state and isomer can be seen in Tables S11–S14. VASP calculations were further completed on the lowest energy isomer, where the magnetic moments are reported in Table S15. In both the fully ligated and undercoordinated NCs, the magnetic moments, calculated with plane wave basis set, match our expectation with the all-electron basis set.

Analyzing the SOMO–LUMO gap of the undercoordinated NCs (Table S16), when M = Cr, Ni, Pd, Al, the gap is larger than that of the parent NC. Unlike the fully ligated NCs, every undercoordinated NC is paramagnetic and hence does not provide a similar explanation for this result. Particularly, Cr is in a very high spin state with a multiplicity of 7 and 6 in the oxidized (2+) NC. There is no common trend across the periods, but the SOMO–LUMO gap gets smaller as the main group metals get bigger down the group.

Reaction 1. [Co_5_MS_8_(PEt_3_)_6_]^1+^ → [Co_5_MS_8_(PEt_3_)_5_]^1+^ + (PEt_3_)_5_ (1)

With the fully ligated and undercoordinated NCs properly modelled, the ligand binding energy was calculated as shown in Table 1 using reaction 1. The explicit mathematical definitions are outlined in the supporting information, and an example is shown in Table S17. It is apparent that all metal dopants in the cationic NCs reduce the ligand binding energy compared to the parent, Co_6_, i.e., incorporating a different metal within the core better promotes ligand loss. In the main group metals, there is a distinct trend that as the metal atom gets bigger (i.e., goes from Al to Ga to In), the ligand binding energy gets smaller. This trend is similar in the 4d metals Mo and Pd; however, the difference is more negligible with a value of only 0.03 eV. There is no common trend with 3d metals. [Co_5_CuS_8_(PEt_3_)_5_]^1+^ has the smallest ligand binding energy, whereas [Co_5_CrS_8_(PEt_3_)_5_]^2+^ has the largest with ΔG_298.15_ values of 0.71 eV and 1.64 eV, respectively. It is notable that every NC prefers a different spin multiplicity in the undercoordinated NCs, compared to its fully ligated counterpart, in the cases of Mn, Fe, Ni, Pd, and the parent Co_6_. This is interesting as the local coordination on the metal atom changed. If this happened in transition metal complexes, we would expect the electronic density to reorient to a different spin state or distort geometrically due to the undercoordinated site. This does not happen in Cu, Mo and Cr NCs. In almost all cases, the LUMO in the L6 NCs have the same molecular orbital as the SOMO of the L5 NC. This molecular orbital consists of a delocalized area contributed from the metal atoms in the core, or a delocalized core with additional density around one ligand. When the ligand is lost, the electron density in the core and around the ligand remains, but additional density localizes on the open metal site. The frontier molecular orbitals for the fully ligated and undercoordinated NCs are shown in Figures S2–S9. There are two exceptions to this trend when M = Mn and Cu. In Mn, there is more local distribution from p atomic orbitals on the sulfur atoms and d atomic orbitals on the open Mn metal in the undercoordinated NC, and there is no distribution on the ligand, which is different in the fully ligated NC. This indicates a back donation from Mn to S p orbitals, demonstrating a redox non-innocent behavior of S. The bonding environment in Cu stays delocalized around the core in the fully ligated NC; however, it becomes more localized on the Cu open site in the undercoordinated NC. The differences between the fully ligated and undercoordinated NCs are difficult to visualize; hence, to further analyze the electronic structures in these NCs and understand the origin of the difference in ligand binding energies from an electronic point of view, detailed natural bond orbital (NBO) analysis was completed.

Using NBO calculations, the natural atomic orbital (NAO) occupation matrix was diagonalized, resulting in d-orbital occupancy as discussed in the Section 2. Despite initial literature hinting that all Co atoms must be in a +2 oxidation state, it is apparent that, regardless of the threshold used, every NC has mixed partial oxidation state (POS), i.e., the six metal atoms hold different oxidation states within each NC, including the parent cluster, [Co_6_S_8_(PEt_3_)_6_]^+^. The POS results can be seen in Tables S18 and S19. Due to this insight, it is better to analyze the same charged cluster to have a direct comparison with how the metal changes the electron density of the NC; hence, we continue our analysis with [Co_5_MS_8_(PEt_3_)_6_]^+^ and [Co_5_MS_8_(PEt_3_)_5_]^+^. Further, considering there is not yet experimentally verified multiplicity in these NCs, it is more meaningful to look at the valence d-orbital occupancy on each metal center. The total alpha and beta d-orbital occupancy can be seen in Tables S20–S23 for the fully ligated NCs, and Tables S24–S27 for the undercoordinated NCs. Overall, Co atoms retain their d-orbital occupancy in the fully ligated NCs with a value on 8.45 ± 0.03 e. There are a few exceptions to this when M = Cr, Al, Ga and In. In the main group metals, the sixth metal (M6) has the lowest Co occupancy, which has the most drastic decrease when M = Al with a value of 8.09 e. In Cr, the lowest Co occupancy is in M4 with a value of 8.16 e. M4 and M6 are both next to the metal dopant (‘cis’ position) which occur in symmetry-equivalent positions of the mirror plane cutting through the dopant atom. With Mn and Fe metals, the ligand is removed from the dopant atom, which is the metal that has the lowest occupancy. Additionally, with Ni and Pd dopants, the ligand is lost from a Co atom, which has a lower d-orbital occupancy than the dopant metal, while having the same occupancy as the other Co atoms in the system. There are three unique cases such that the ligand is removed from the metal that does not have the lowest occupancy, i.e., M = Cr, Mo, and Cu. In Cr and Mo, the metal dopant has lower occupancy than the Co atoms; however, the ligand is lost from one of the Co atoms. For Cu, the Co atoms have less occupancy; however, the ligand is removed from Cu atom. Recall that Cu has the lowest binding energy, meaning that it is the most favorable to ligand loss. This hints that the ligand binding energy is reduced when the ligand is removed from the most electronically donating metal. In the undercoordinated NCs, there is no electronic occupancy change for Cr, Ni, Cu, and Pd NCs in the dopant atom. The localized change from ligand loss in these NCs is only shown in the Co atoms. Specifically, the Co atoms are lower in occupancy in M1 and M6 when M = Cr, M2 when M = Ni and Pd, and M4 when M = Cu. In Cu, and Cr, M1, M4 and M6 are the Co atoms right next to the dopant in symmetry equivalent positions across the mirror plane. In Ni and Pd, the ligand is removed from M2. Recall that Ni and Pd have the largest ligand binding energy, meaning they have the most preference to the fully ligated NC. Considering the Co atom that loses the ligand has the least occupancy and all the other metals in the system remain unchanged, this again hints that ligand binding energy can be reduced if the ligand lost is from the most electron donating metal. Incorporation with the Fe atom is unique in that the Co atoms remain unchanged in both undercoordinated and fully ligated NCs. The occupancy is only changed within the d-orbitals on Fe. This was observed in our previous study [11].

As discussed in the introduction, the undercoordinated NCs can be deposited onto SAM surfaces, giving well-defined undercoordinated NCs that stay intact on the surface [12]. While studies of the deposition mechanism are being studied, if we assume that the open metal center is accessible for catalysis, these computations could serve as a vital model to test catalytic reactivity. To analyze this, the reaction pathways for HER and CO_2_R to CO were completed on the undercoordinated NCs using the computational hydrogen electrode (CHE) model [39,40].

**Figure 2:**
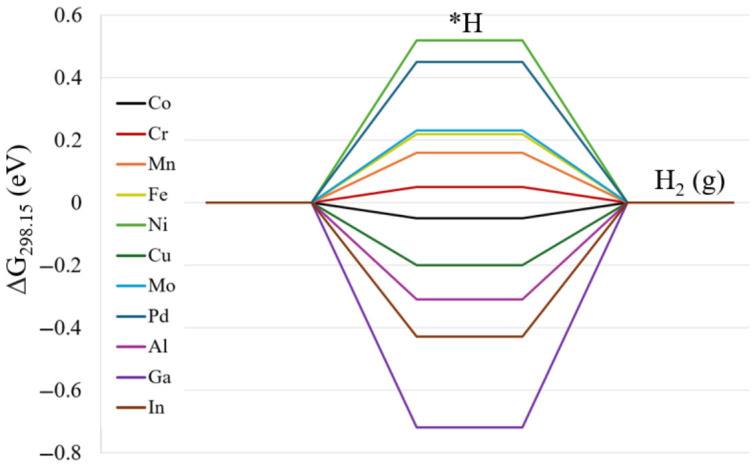
Reaction energy diagram of HER using the CHE model (reaction 2 and 3) for [Co_5_MS_8_(PEt_3_)_5_]^1+^ NCs at the PBE0-D3/def2TZVP(opt)/CEP-31G(freq) level of theory.

Initially, HER was analyzed following reaction 2 and 3 with the NC ([Co_5_MS_8_(PEt_3_)_5_]^n+^) as the reference state. The hydrogen adsorbed NC, [Co_5_MS_8_(PEt_3_)_5_H]^n+^ (*H) is assumed as the intermediate for HER. This intermediate was analyzed by testing three different binding sites: the sulfur atom, the metal atom, and the ‘bridge’ site between two metal atoms as depicted in Figure S10. An additional site was calculated for the undercoordinated NCs, in which the ligand prefers to be removed from a cobalt atom next to the dopant metal (Co-cis configuration) due to symmetry. Specifically, for the sulfur active site we considered the sulfur next to the metal dopant as well as the sulfur on the cobalt next to other cobalt atoms (‘S1’ and ‘S2’, respectively, Figure S10). The relative energies of different spin multiplicities and isomers can be seen in Tables S28–S31. Regardless of the metal atom incorporated within our subset, the sulfur atom is found to be the preferred H binding site with the lowest energy of *H. Figure 2 shows the HER free energy profiles at 298.15K for each NC. Out of all possible metal incorporations, the systems that are the most efficient for HER are Cr doped cluster and the parent Co cluster with H adsorption energies of 0.05 eV, and −0.05 eV, respectively. Ga overbinds H with a value of −0.72 eV, leaving a large uphill reaction free energy for H_2_. On the other hand, Ni and Pd binding with H are the most energetically uphill (0.52 eV and 0.45 eV, respectively). These endergonic H adsorptions, along with other uphill H adsorptions in Mn, Fe, and Mo could potentially be overcome upon photoexcitation of the catalyst, which can be of interest in future studies. The reaction energies of hydrogen adsorption for all metals can be seen in Table S31.

Reaction 2. [Co_5_MS_8_(PEt_3_)_5_]^n+^ + 0.5H_2_ → [Co_5_MS_8_(PEt_3_)_5_H]^n+^ (*H); n = 1 or 2 (2)

Reaction 3. [Co_5_MS_8_(PEt_3_)_5_H]^n+^ (*H) + 0.5H_2_ → [Co_5_MS_8_(PEt_3_)_5_]^n+^ + H_2_ (g) (3)

**Figure 3:**
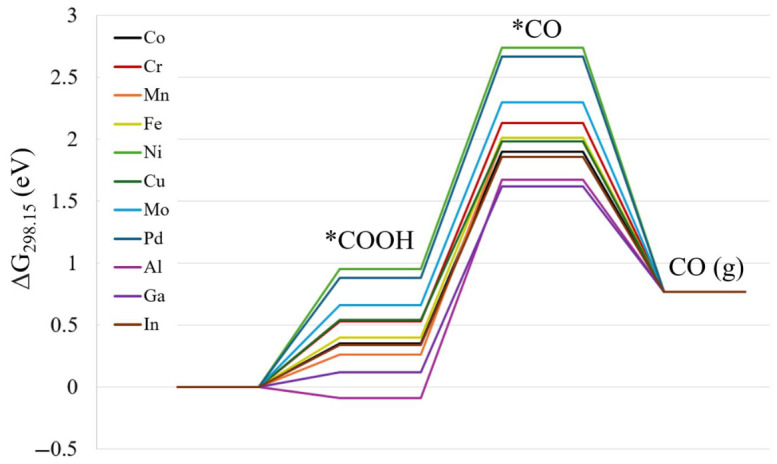
Reaction energy diagram of CO_2_ reduction using the CHE model (reaction S2–S4) for [Co_5_MS_8_(PEt_3_)_5_]^1+^ NCs at the PBE0-D3/def2TZVP(opt)/CEP-31G(freq) level of theory.

In addition to HER, CO_2_R to CO was analyzed using the CHE model. In the parent and metal dopant NCs, CO_2_ does not bind to the NC, and it comes off during optimization. However, the first possible intermediates, HCOO* and *COOH, both bind to the open site in the NCs. Like *H, *HCOO and *COOH configurations were calculated by testing three different sites: the sulfur atom, the metal atom, and the ‘bridge’ site between two metal atoms, as depicted by the *COOH configurations in Figure S11. The relative energies of different spin multiplicities and isomers are shown in Tables S33–S38. In each case, the NC prefers COOH binding on sulfur atoms, i.e., the lowest energy conformation. It is noted that sulfur is the preferred binding site for small molecule adsorption in these NCs. Reaction energies for *COOH are shown in Table S39. Following the *COOH mechanism for CO_2_ reduction, CO is the second intermediate. While CO should keep the same spin multiplicity as the NC catalyst, other spin multiplicities were checked just in case as shown in Table S40. It is notable that since the sulfur atom is the lowest energy-active site, *CO binds molecularly within the sterically free area, rather than CO attached to the metal like a ligand. This structure is different than CO ligand exchange mechanisms reported with these NCs in the literature [41]. The reaction energies of *CO and CO(g) production are shown in Tables S41 and S42, and the reaction energy diagram for CO_2_ reduction is shown in Figure 3. In every case, CO_2_R to CO has calculated reaction free energy of 0.78 eV. While most *COOH formation energies are uphill, it is actually *CO formation from COOH* that is the rate-limiting step for CO_2_R to CO in these NCs. Although photoexcitation of the catalyst may lower the energy needed to form COOH*, this energy required for CO* formation still needs high electrical voltage, hinting that most of these NCs might not make good photocatalysts or electrocatalysts. After analyzing the energy profiles, the only systems which may be electrocatalytically efficient for CO_2_ reduction are [Co_5_MS_8_(PEt_3_)_5_]^1+^ (M = Al, Ga, and In).

**Figure 4:**
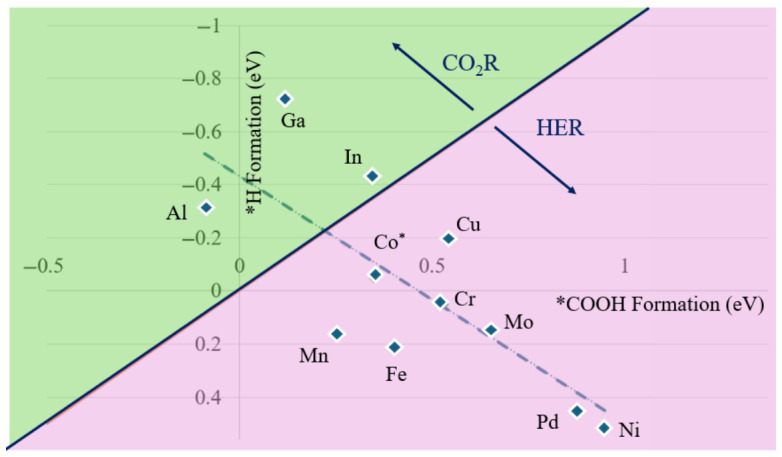
*H formation energy as a function of *COOH formation energy on the sulfur site for [Co_5_MS_8_(PEt_3_)_5_]^1+^ NCs at the PBE0-D3/def2TZVP(opt)/CEP-31G(freq) level of theory. The green area is more selective towards CO_2_ reduction, while the pink area is more selective towards HER (* = parent NC, linear regression: y = 0.9288x − 0.4317, R^2^ = 0.59).

HER is known to compete with CO_2_R under reaction conditions, which drastically changes catalytic selectivity. For example, recent Cu catalysts have shown to suppress HER as the adsorbed CO intermediate resides on the active site [42,43]. For transition metal NC based electrocatalytic studies, H* and *COOH formation energy are often the limiting potentials for CO_2_R and HER [16]. Considering that under reaction conditions, the NC would competitively prefer H* or *COOH, we apply the difference in limiting potentials between these two reaction steps as the qualitative descriptor for selectivity. This has been previously done in Rybacki, M. et al.’s article where they mapped out the competition between HER and CO_2_R in Au_24_M NCs [16]. Using this method, a positive difference between CO_2_R and HER implies selectivity towards CO(g) formation as opposed to H_2_(g) formation. Comparing ligand removal energy and *COOH formation energy (and similarly with H* formation energy), we obtain a weak linear behavior (R^2^ = 0.59) between *COOH and H* formation energy as seen in Figure 4. In Figure 4, the green and pink areas signify the NCs that prefer CO(g) formation or H_2_(g) production, respectively. These areas are calculated by implying that a positive energy difference of CO_2_ reduction from HER promotes CO(g). Notably, these do not account for the next step of CO_2_R, or the conversion from *COOH to *CO. The calculated energy differences are shown in Figure S12. Note that the negative axis for *H formation stems from the linear relationship between the work function and energy differences, which relates to the HER volcano and Sabatier principle (i.e., an ideal HER catalyst should have a formation energy close to 0 eV) [44,45]. This clearly shows that the choice of dopant significantly influences the selectivity of the catalyst. Notably, most of the NCs competitively prefer HER, except main group metals. It is further notable that this weak linear relationship between HER and CO_2_R is held between heteroatom dopants in noble metal atomically precise Au_24_ NCs (R^2^ = 0.60) NC. This hints that, while different metallically, atomically precise NCs that show superatomic nature may share similar relationships, not only between structural changes and motifs, but with preferred active sites and selectivity.

**Figure 5:**
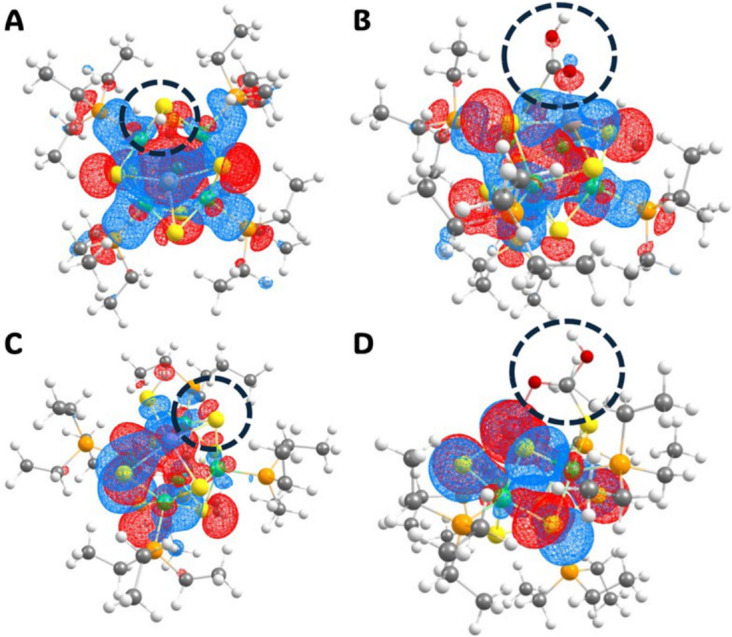
Pictures of the singly occupied molecular orbital (SOMO) for H* and *COOH structures at the PBE0-D3/def2TZVP level of theory for [Co_5_MS_8_(PEt_3_)_5_]^1+^ NCs. Specifically, (**A**) [Co_5_AlS_8_(PEt_3_)_5_H]^1+^ (**B**) [Co_5_AlS_8_(PEt_3_)_5_COOH]^1+^ (**C**) [Co_5_MnS_8_(PEt_3_)_5_H]^1+^, and (**D**) [Co_5_MnS_8_(PEt_3_)_5_COOH]^1+^. It is notable that when M = Al, CO_2_R is the preferred pathway, whereas when M = Mn, HER is the favored pathway. The black dashed rings denote the adsorbed molecule/atom. Contour = 0.02.

With NBO analysis, it is apparent that the bonding environment in the main group metals is different than the transition metal atoms in the occupied MOs. Specifically, there is a large contribution on the *p* atomic orbitals in the oxygen atoms on *COOH as well as the open metal site. There is no contribution on the sulfur or the hydrogen for *H structures, so the density is solely delocalized within the core. This is the opposite for the dopants that prefer HER. Specifically, there is no density on the occupied MOs of the *COOH species with NBO. However, in *H there is large contribution on the *p* orbitals from the sulfur atoms. A perfect example of this is shown in Figure 5, where the SOMO in [Co_5_AlS_8_(PEt_3_)_5_H]^1+^ has no contribution to the adsorbed hydrogen from the sulfur site atom, but large contribution from the sulfur and oxygen atoms in [Co_5_AlS_8_(PEt_3_)_5_COOH]^1+^. When M = Mn, however, the opposite trend happens. From our linear relationship results, we know Al is more active toward CO_2_R, and Mn is more active toward HER. Further, it is notable that the geometry drastically changes in the *H species with the main group metals. The sulfur–metal dopant bond breaks and largely increases distance so that the dopant can retain three coordination sites, while the sulfur retains the hydrogen. This does not happen in the *COOH species, while the COOH molecule stays bonded to the sulfur, it optimizes diagonally so that the oxygen atoms are bridging the metals.

It is critical to further comment on the importance of the binding site. In every optimized *H or *COOH NC within our subset, it was apparent that the preferred binding site is with the sulfur atom regardless of the heteroatom. While transition metals are typically the redox-active site in HER and CO_2_R, our study is not the first to observe such phenomenon. For instance, Rybacki et al. computationally studied Au_24_M NCs for HER and CO_2_R electrocatalysis and analyzed the active site for H binding. They also found that the H binds onto the S site of the clusters instead of the metals [16]. In addition, a previous study by Niklas et al. reported HER using cobaloximes and cobalt poly(pyridyl) complexes. In this study they used multifrequency electron paramagnetic resonance (EPR) spectroscopy (X-band, Q-band, and D-band) in combination with DFT modeling to elucidate the electronic structure of three Co(II) molecular catalysts for HER and discovered that H binds onto the N on the ligand instead of Co center [46]. While our calculations are limited to adsorption thermodynamics, providing us only a small picture of the dynamics without detailed evaluation of the transition states or alternative reaction pathways, our data suggests that further studies of H binding sites of NCs and metal complexes for HER should take into account both the metal centers and their nearby main group atoms.

For completion, HER and CO_2_R were also completed on the [Co_5_MS_8_(PEt_3_)_5_]^2+^ (M = Cr, Al, Ga, In) NCs. Recall that the NCs have mixed oxidation state and therefore the cluster does not need to be fully oxidized (+2 charge state) to incorporate Cr, Al, Ga and In metals into the system. These NCs were analyzed for completion in case they experimentally change the overall oxidation state of the NC. Each NC still prefers the sulfur-binding site; however, the bonding environment is drastically distorted upon adsorption in both *H and *COOH geometries. Specifically, the sulfur site will come unbonded to the metal, creating a tetrahedral formation with the PEt_3_ ligand, while the undercoordinated site only slightly changes to make room for the bonded H or COOH molecule (Figure S13). It is notable that this charge state does not improve the catalytic efficiency HER on the NCs as seen in the energy diagram for HER (Figure S14). For CO_2_R, the *COOH barrier is slightly reduced with NCs at this charge state; however, *CO adsorption still leads to a very large barrier, making efficient CO_2_ reduction unlikely (Figure S15).

## 4. Conclusions

In this paper, the electronic structures and catalytic properties of the undercoordinated hexanuclear cobalt sulfide NCs were studied. Initially, the effect of heteroatom incorporation on the geometry of the fully ligated and undercoordinated NCs were analyzed at different spin multiplicities. The reactivities of ten different metals were analyzed. While there is not a dominate pattern as these NCs locally change in response to their mono-dopant, this work shows that the type of metal incorporation greatly affects the electronic structure and formation energies for ligand binding and catalysis. Particularly, the d-orbital occupancy in the cobalt atoms remains largely unchanged, while the heteroatom greatly influences the reactivity of the undercoordinated NCs. All metal dopants in the cationic NCs reduce the ligand binding energy compared to the parent, Co_6_, i.e., incorporating a different metal within the core will better promote ligand loss. Further, ligand binding energy can be reduced if the ligand lost is from the most electron donating metal. Most notably, this work highlights that transition metal incorporated [Co_5_MS_8_(PEt_3_)_5_]^1+^ would competitively prefer HER over CO_2_R, while the main group metal dopants prefer CO_2_R. Regardless of the metal atom incorporated, the sulfur atom is the preferred binding site for small molecule adsorption in all NCs. This work illustrates the electronic structures and catalytic properties of the undercoordinated hexanuclear cobalt sulfide NCs, providing molecular-level understandings in predictive design of these types of NCs for catalytic HER and CO_2_R.

## Figures and Tables

**Table 1:** Ligand binding energies calculated using reaction 1 at the PBE0-D3/def2TZVP(opt)/CEP-31G(freq) level of theory for all [Co_5_MS_8_(PEt_3_)_5_]^1+/2+^ NCs in gas phase, with a zero-point energy correction, and with a Gibbs correction at 298.15K. The details for the calculation are described in the supporting information. (C/M = Charge/multiplicity, ZPE = Zero-point energy.)

	C/M-L6	C/M-L5	Isomer	ΔE (eV)	ΔE_ZPE_ (eV)	ΔG_298.15_ (eV)
Co	1/2	1/4	Co	2.24	2.16	1.53
Cr	1/5, 2/6	1/7, 2/6	Co-Cis, Co-Cis	2.06, 2.39	1.97, 2.25	1.23, 1.64
Mn	1/4	1/6	Mn	1.96	1.88	1.20
Fe	1/3	1/5	Fe	2.00	1.93	1.29
Ni	1/1	1/3	Co-Trans	2.17	2.06	1.37
Cu	1/2	1/2	Cu	1.49	1.44	0.71
Mo	1/3	1/3	Co-Cis	2.16	2.08	1.40
Pd	1/1	1/3	Co-Trans	2.18	2.08	1.37
Al	1/2, 2/3	1/2, 2/3	Al, Co-Cis	1.86, 2.30	1.82, 2.20	1.14, 1.53
Ga	1/2, 2/3	1/2, 2/5 *	Ga, Co-Cis	1.62, 2.22	1.60, 2.08	1.01, 1.46
In	1/2, 2/3	1/2, 2/3	In, Co-Cis	1.49, 2.13	1.49, 2.02	0.82, 1.38

* Note: Isomer of Ga with 2/3 C/M is only 2.7 × 10^−5^ eV higher in energy.

## Data Availability

The original contributions presented in this study are included in the article/Supplementary Material. Further inquiries can be directed to the corresponding author.

## Supplementary Materials

The following supporting information can be downloaded at: https://www.mdpi.com/article/10.3390/nano16100587/s1, Table S1. Summary table of the lowest energy spin multiplicity for [Co_6−x_M_x_S_8_(PEt_3_)_6_]^n+^ (x=1–3, n = 0, 1, 2) NCs at the PBE0-D3/def2TZVP level of theory (* = parent cluster); Table S2. Relative energies at different spin multiplicities and symmetry isomers for [Co_6−x_M_x_S_8_(PEt_3_)_6_]^1+^ (x = 1–3, M = Co, Cr, Mn) NCs at the PBE0-D3/def2TZVP level of theory (C/M = Charge/Multiplicity); Table S3. Relative energies at different spin multiplicities and symmetry isomers for [Co_6–x_M_x_S_8_(PEt_3_)_6_]^1+^ (x = 1–3, M = Fe, Ni, Cu) NCs at the PBE0-D3/def2TZVP level of theory (C/M = Charge/Multiplicity); Table S4. Relative energies at different spin multiplicities and symmetry isomers for [Co_6−x_M_x_S_8_(PEt_3_)_6_]^1+^ (x = 1–3, M = Mo, Pd) NCs at the PBE0-D3/def2TZVP level of theory (C/M = Charge/Multiplicity); Table S5. Relative energies at different spin multiplicities and symmetry isomers for [Co_6-x_M_x_S_8_(PEt_3_)_6_]^1+^ (x = 1–3, M = Al, Ga, In) NCs at the PBE0-D3/def2TZVP level of theory (C/M = Charge/Multiplicity); Table S6. Relative energies at different spin multiplicities for [Co_5_MS_8_(PEt_3_)_6_]^2+^ (M = Al, Ga, In, Cr) NCs at the PBE0-D3/def2TZVP level of theory (C/M = Charge/Multiplicity); Table S7. Magnetic moment values (calculated using VASP) and the lowest energy spin multiplicity (calculated using ORCA) for [Co_5_MS_8_(PEt_3_)_6_]^1+/2+^ NCs (* = parent cluster); Table S8. SOMO-LUMO Gaps (eV) for [Co_6−x_M_x_S_8_(PEt_3_)_6_]^n+^ (x = 1–3, n = 0, 1, 2) NCs at the PBE0-D3/def2TZVP level of theory (* = parent cluster); Table S9. Average bond distances (Å) for [Co_5_MS_8_(PEt_3_)_6_]^1+^ NCs in their lowest energy spin multiplicity the PBE0-D3/def2TZVP level of theory (* = parent cluster); Table S10. Summary table of the lowest energy spin multiplicity for [Co_5_MS_8_(PEt_3_)_5_]^n+^ (n = 1, 2) NCs at the PBE0-D3/def2TZVP level of theory (* = parent cluster); Table S11. Relative energies (eV) at different spin multiplicities and symmetry isomers (Conf) for [Co_5_MS_8_(PEt_3_)_5_]^1+^ (M = Co, Cr, Mn) NCs at the PBE0-D3/def2TZVP level of theory (C/M = Charge/Multiplicity); Table S12. Relative energies at different spin multiplicities and symmetry isomers (Conf) for [Co_5_MS_8_(PEt_3_)_5_]^1+^ (M = Mo, Pd) NCs at the PBE0-D3/def2TZVP level of theory (C/M = Charge/Multiplicity); Table S13. Relative energies (eV) at different spin multiplicities and symmetry isomers (Conf) for [Co_5_MS_8_(PEt_3_)_5_]^1+^ (M = Al, Ga, In) NCs at the PBE0-D3/def2TZVP level of theory (C/M = Charge/Multiplicity); Table S14. Relative energies at different spin multiplicities and symmetry isomers (Conf) for [Co_5_MS_8_(PEt_3_)_5_]^2+^ (M = Al, Ga, In, Cr) NCs at the PBE0-D3/def2TZVP level of theory (C/M = Charge/Multiplicity); Table S15. Magnetic moment values (calculated using VASP) and the lowest energy spin multiplicity (calculated using ORCA) for [Co_5_MS_8_(PEt_3_)_5_]^1+/2+^ NCs (* = parent cluster); Table S16. SOMO–LUMO Gaps (eV) for [Co_5_MS_8_(PEt_3_)_6_]^n+^ (n = 0, 1, 2) NCs at the PBE0-D3/def2TZVP level of theory (* = parent cluster); Table S17. Example of the Zero-point energy and Gibbs free energy corrections for the calculation of ligand binding in Co_6−x_Fe_x_ (x = 1, 3) NCs at the PBE0-D3/def2TZVP (ORCA – geometry optimizations) and PBE0-D3/CEP-31G (Gaussian - single point calculations) level of theory. All values are shown in Hartree; Table S18. POS value at ‘0.65’ value threshold for [Co_5_MS_8_(PEt_3_)_6_]^1+^ NCs (* = parent cluster) at the lowest spin multiplicity; Table S19. POS value at ‘0.65’ value threshold for [Co_5_MS_8_(PEt_3_)_5_]^1+^ NCs (* = parent cluster) at the lowest spin multiplicity; Table S20. Total alpha and beta d-orbital occupancy for each metal center in [Co_5_MS_8_(PEt_3_)_6_]^1+^ (M = Co, Cr, Mn) NCs at the lowest spin multiplicity (pink = dopant); Table S21. Total alpha and beta d-orbital occupancy for each metal center in [Co_5_MS_8_(PEt_3_)_6_]^1+^ (M = Fe, Ni, Cu) NCs at the lowest spin multiplicity (pink = dopant); Table S22. Total alpha and beta d-orbital occupancy for each metal center in [Co_5_MS_8_(PEt_3_)_6_]^1+^ (M = Mo, Pd) NCs at the lowest spin multiplicity (pink = dopant); Table S23. Total alpha and beta d-orbital occupancy for each metal center in [Co_5_MS_8_(PEt_3_)_6_]^1+^ (M = Al, Ga, In) NCs at the lowest spin multiplicity; Table S24. Total alpha and beta d-orbital occupancy for each metal center in [Co_5_MS_8_(PEt_3_)_5_]^1+^ (M = Co, Cr, Mn) NCs at the lowest spin multiplicity (pink = dopant); Table S25. Total alpha and beta d-orbital occupancy for each metal center in [Co_5_MS_8_(PEt_3_)_5_]^1+^ (M = Fe, Ni, Cu) NCs at the lowest spin multiplicity (pink = dopant); Table S26. Total alpha and beta d-orbital occupancy for each metal center in [Co_5_MS_8_(PEt_3_)_5_]^1+^ (M = Mo, Pd) NCs at the lowest spin multiplicity (pink = dopant); Table S27. Total alpha and beta d-orbital occupancy for each metal center in [Co_5_MS_8_(PEt_3_)_5_]^1+^ (M = Al, Ga, In) NCs at the lowest spin multiplicity (pink = dopant); Table S28. Relative energies (eV) at different spin multiplicities and symmetry isomers (Conf) for *H adsorption or [Co_5_MS_8_(PEt_3_)_5_H]^1+^ (M = Co, Cr, Mn, Fe, Ni, Cu) NCs at the PBE0-D3/def2TZVP level of theory (C/M = Charge/Multiplicity); Table S29. Relative energies (eV) at different spin multiplicities and symmetry isomers (Conf) for *H adsorption or [Co_5_MS_8_(PEt_3_)_5_H]^1+^ (M = Mo, Pd) NCs at the PBE0-D3/def2TZVP level of theory (C/M = Charge/Multiplicity); Table S30. Relative energies (eV) at different spin multiplicities and symmetry isomers (Conf) for *H adsorption or [Co_5_MS_8_(PEt_3_)_5_H]^1+^ (M = Al, Ga, In) NCs at the PBE0-D3/def2TZVP level of theory (C/M = Charge/Multiplicity); Table S31. Relative energies (eV) at different spin multiplicities and symmetry isomers (Conf) for *H adsorption or [Co_5_MS_8_(PEt_3_)_5_H]^2+^ (M = Al, Ga, In) NCs at the PBE0-D3/def2TZVP level of theory (C/M = Charge/Multiplicity); Table S32. Reaction energies for hydrogen adsorption following reaction 1, n = 1 (C/M = Charge/multiplicity, ZPE = Zero-point energy); Table S33. Relative energies (eV) at different spin multiplicities and symmetry isomers (Conf) for the first intermediate of CO_2_ reduction (*COOH or HCOO*) with M = Co, Cr, Mn NCs at the PBE0-D3/def2TZVP level of theory (C/M = Charge/Multiplicity); Table S34. Relative energies (eV) at different spin multiplicities and symmetry isomers (Conf) for the first intermediate of CO_2_ reduction (*COOH or HCOO*) with M = Fe, Ni, Cu NCs at the PBE0-D3/def2TZVP level of theory (C/M = Charge/Multiplicity); Table S35. Relative energies (eV) at different spin multiplicities and symmetry isomers (Conf) for the first intermediate of CO_2_ reduction (*COOH or HCOO*) with M = Mo, Pd NCs at the PBE0-D3/def2TZVP level of theory (C/M = Charge/Multiplicity); Table S36. Relative energies (eV) at different spin multiplicities and symmetry isomers (Conf) for the first intermediate of CO_2_ reduction (*COOH or HCOO*) with M = Al, Ga, In NCs at the PBE0-D3/def2TZVP level of theory (C/M = Charge/Multiplicity); Table S37. Relative energies (eV) at different spin multiplicities and symmetry isomers (Conf) for the first intermediate of CO_2_ reduction (*COOH or HCOO*) with M = Al, Ga NCs at the PBE0-D3/def2TZVP level of theory (C/M = Charge/Multiplicity); Table S38. Relative energies (eV) at different spin multiplicities and symmetry isomers (Conf) for the first intermediate of CO_2_ reduction (*COOH or HCOO*) with M = In, Cr NCs at the PBE0-D3/def2TZVP level of theory (C/M = Charge/Multiplicity); Table S39. Reaction energies for COOH adsorption following reaction S2, n = 1 (C/M = Charge/multiplicity, ZPE = Zero-point energy); Table S40. Relative energies (eV) at different spin multiplicities for the second intermediate of CO_2_ reduction (*CO) with M = Co, Fe, Cu, Al NCs at the PBE0-D3/def2TZVP level of theory (C/M = Charge/Multiplicity); Table S41. Reaction energies for CO adsorption following reaction S3, n = 1 (C/M = Charge/multiplicity, ZPE = Zero-point energy); Table S42. Reaction energies for the release of CO(g) following reaction S4, n = 1 (C/M = Charge/multiplicity, ZPE = Zero-point energy); Table S43. Energy calculation for [Co_5_MS_8_(PEt_3_)_5_]^0^ (M = Co, Fe, Mn, Al) NCs at the PBE0-D3/def2TZVP level of theory (C/M = Charge/Multiplicity); Figure S1. Symmetry isomers of undercoordinated Co_5_Fe NCs (A) Co – trans, (B) ‘M’, M = Cr, Mn, Fe, Ni, Cu, Mo, Pd, Al, Ga, In and (C) Co – cis; Figure S2. SOMO/LUMO molecular orbitals of [Co6S_8_(PEt_3_)_x_]^0/1+^ at PBE0-D3/def2TZVP(opt)/CEP-31G(freq) level of theory; Figure S3. SOMO/LUMO molecular orbitals of [Co_5_CrS_8_(PEt_3_)_x_]^1+/2+^ at PBE0-D3/def2TZVP(opt)/CEP-31G(freq) level of theory; Figure S4. SOMO/LUMO molecular orbitals of [Co_5_MS_8_(PEt_3_)_x_]^1+^ (M = Mn, Fe) at PBE0-D3/def2TZVP(opt)/CEP-31G(freq) level of theory; Figure S5. SOMO/LUMO molecular orbitals of [Co_5_MS_8_(PEt_3_)_x_]^1+^ (M = Ni, Cu) at PBE0-D3/def2TZVP(opt)/CEP-31G(freq) level of theory; Figure S6. SOMO/LUMO molecular orbitals of [Co_5_MS_8_(PEt_3_)_x_]^1+^ (M = Mo, Pd) at PBE0-D3/def2TZVP(opt)/CEP-31G(freq) level of theory; Figure S7. SOMO/LUMO molecular orbitals of [Co_5_AlS_8_(PEt_3_)_x_]^1+/2+^ at PBE0-D3/def2TZVP(opt)/CEP-31G(freq) level of theory; Figure S8. SOMO/LUMO molecular orbitals of [Co_5_GaS_8_(PEt_3_)_x_]^1+/2+^ at PBE0-D3/def2TZVP(opt)/CEP-31G(freq) level of theory; Figure S9. SOMO/LUMO molecular orbitals of [Co_5_InS_8_(PEt_3_)_x_]^1+/2+^ at PBE0-D3/def2TZVP(opt)/CEP-31G(freq) level of theory; Figure S10. Initial starting geometries for *H species in each NC. (A) S1—Sulfur next to metal dopant, (B) S2—Sulfur next to cobalt across from metal dopant, (C) Bridged site between two metal atoms, (D) Directly on the metal atom; Figure S11. Initial starting geometries for *COOH species (circled) in each NC. (A) S1—Sulfur next to metal dopant, (B) S2—Sulfur next to cobalt across from metal dopant, (C) Bridged site between two metal atoms, (D) Directly on the metal atom; Figure S12. Difference in reaction energy of *COOH vs. *H formation on the S active sites for [Co_5_MS8(PEt3)5]1+ NCs at the PBE0-D3/def2TZVP(opt)/CEP-31G(freq) level of theory. Positive values reflect selectivity towards CO (g) formation while negative values reflect selectivity towards H_2_ (g) formation; Figure S13. Pictorial image of bonding environment upon small molecule adsorption in [Co_5_MS_8_(PEt_3_)_5_]^2+^ NCs; Figure S14. Reaction energy diagram of HER for [Co_5_MS_8_(PEt_3_)_5_]^2+^ NCs at the PBE0-D3/def2TZVP(opt)/CEP-31G(freq) level of theory; Figure S15. Reaction energy diagram of CO_2_ reduction for [Co_5_MS_8_(PEt_3_)_5_]^2+^ NCs at the PBE0-D3/def2TZVP(opt)/CEP-31G(freq) level of theory.
