# Supplementary material for: Exploration of the Electronic and Catalytic Properties of [Co5MS8(PEt3)5]1+ Nanoclusters: A Computational Study
Source: Nanomaterials (Basel). 2026 May 12;16(10):587. doi: 10.3390/nano16100587 (PMC13209726; doi:10.3390/nano16100587)
Supplement: Supplementary file 1 [file nanomaterials-16-00587-s001.zip › nanomaterials-4218155-supplementary.pdf]

Exploration of the Electronic and Catalytic Properties of $[Co_5MS_8(PEt_3)_5]^{1+}$ Nanoclusters: A Computational Study

Shana Havenridge[1], Audrey Grace Miller[2], and Cong Liu[1*]

1. Chemical Sciences and Engineering Division, Argonne National Laboratory, Lemont, IL 60439, USA.
2. Illinois Mathematics and Science Academy, Aurora, IL 60506, USA

*Corresponding author email: congliu@anl.gov

Supporting Information

Fully Ligated (L_6) Nanoclusters:

Table S1. Summary table of the lowest energy spin multiplicity for $[Co_{6-x}M_xS_8(PEt_3)_6]^{n+}$ (x=1-3, n = 0, 1, 2) NCs at the PBE0-D3/def2TZVP level of theory (* = parent cluster).

Metal	Charge of NC	x=1	x=2	x=3
Co*	0/1+	1/2	--	--
Cr	1+/2+	5/6	2 - cis	5
Mn	1+	4	2 - cis	2
Fe	1+	3	--	3
Ni	1+	1	2 - trans	3
Cu	1+	2	--	2
Mo	1+	3	2 - cis	1
Pd	1+	1	2 - cis	3
Al	1+/2+	2/3	2 - cis	2
Ga	1+/2+	2/3	2 - cis	2
In	1+/2+	2/3	2 - cis	2

Table S2. Relative energies at different spin multiplicities and symmetry isomers for $[Co_{6-x}M_xS_8(PEt_3)_6]^{1+}$ (x=1-3, M=Co, Cr, Mn) NCs at the PBE0-D3/def2TZVP level of theory (C/M = Charge/Multiplicity).

(eV)	C/M	Co	C/M	Co	C/M	Cr	C/M	Mn
1	**0/1**	**0.00**	**1/2**	**0.00**	1/1	1.74	1/2	1.22
	0/3	0.72	1/4	0.66	1/3	0.002	**1/4**	**0.00**
	0/5	1.40	1/6	1.08	**1/5**	**0.000**	1/6	0.59
	--	--	--	--	1/7	0.90	--	--
2a - 'cis'	--	--	--	--	**1/2**	**0.00**	**1/2**	**0.00**
	--	--	--	--	1/4	0.84	1/4	1.09
	--	--	--	--	1/6	0.01	1/6	0.42
2b - 'trans'	--	--	--	--	1/2	SCF	1/2	0.60
	--	--	--	--	1/4	SCF	1/4	SCF
	--	--	--	--	1/6	0.11	1/6	0.68
3	--	--	--	--	1/1	3.43	**1/2**	**0.00**
	--	--	--	--	1/3	0.002	1/4	0.56
	--	--	--	--	**1/5**	**0.000**	1/6	1.11
	--	--	--	--	1/7	0.92	--	--

Table S3. Relative energies at different spin multiplicities and symmetry isomers for $[Co_{6-x}M_xS_8(PEt_3)_6]^{1+}$ (x=1-3, M=Fe, Ni, Cu) NCs at the PBE0-D3/def2TZVP level of theory (C/M = Charge/Multiplicity).

(eV)	C/M	Fe	C/M	Ni	C/M	Cu
1	1/1	1.36	**1/1**	**0.00**	**1/2**	**0.00**
	**1/3**	**0.00**	1/3	0.59	1/4	0.28
	1/5	0.34	1/5	1.40	1/6	0.67
	--	--	--	--	--	--
2a - 'cis'	--	--	1/2	0.11	--	--
	--	--	1/4	SCF	--	--
	--	--	1/6	1.20	--	--
2b - 'trans'	--	--	**1/2**	**0.00**	--	--
	--	--	1/4	0.60	--	--
	--	--	1/6	SCF	--	--
3	1/1	2.50	1/1	0.84	**1/2**	**0.00**
	**1/3**	**0.00**	**1/3**	**0.00**	1/4	SCF
	1/5	0.03	1/5	0.60	1/6	0.07
	1/7	0.07	--	--	--	--

Table S4. Relative energies at different spin multiplicities and symmetry isomers for $[Co_{6-x}M_xS_8(PEt_3)_6]^{1+}$ (x=1-3, M=Mo, Pd) NCs at the PBE0-D3/def2TZVP level of theory (C/M = Charge/Multiplicity).

(eV)	C/M	Mo	C/M	Pd
1	1/1	0.52	**1/1**	**0.00**
	**1/3**	**0.00**	1/3	0.66
	1/5	0.46	1/5	1.25
	--	--	--	--
2a - 'cis'	**1/2**	**0.00**	**1/2**	**0.00**
	1/4	0.20	1/4	0.67
	1/6	0.91	1/6	1.14
2b - 'trans'	1/2	0.23	1/2	0.27
	1/4	0.46	1/4	0.23
	1/6	0.87	1/6	1.57
3	**1/1**	**0.00**	1/1	0.34
	1/3	0.02	**1/3**	**0.00**
	1/5	1.23	1/5	0.60
	--	--	--	--

Table S5. Relative energies at different spin multiplicities and symmetry isomers for $[Co_{6-x}M_xS_8(PEt_3)_6]^{1+}$ (x=1-3, M=Al, Ga, In) NCs at the PBE0-D3/def2TZVP level of theory (C/M = Charge/Multiplicity).

(eV)	C/M	Al	C/M	Ga	C/M	In
1	**1/2**	**0.00**	**1/2**	**0.00**	**1/2**	**0.00**
	1/4	0.46	1/4	0.24	1/4	0.54
	1/6	1.26	1/6	0.98	1/6	0.95
	--	--	--	--	--	--
2a - 'cis'	**1/2**	**0.00**	**1/2**	**0.00**	**1/2**	**0.00**
	1/4	0.47	1/4	0.41	1/4	0.36
	1/6	0.69	1/6	0.55	1/6	0.80
2b - 'trans'	1/2	0.13	1/2	0.17	1/2	0.33
	1/4	0.61	1/4	0.63	1/4	0.77
	1/6	0.78	1/6	0.94	1/6	0.33
3	**1/2**	**0.00**	**1/2**	**0.00**	**1/2**	**0.00**
	1/4	0.32	1/4	0.31	1/4	0.27
	1/6	0.72	1/6	0.56	1/6	0.21
	--	--	--	--	--	--

Table S6. Relative energies at different spin multiplicities for $[Co_5MS_8(PEt_3)_6]^{2+}$ (M=Al, Ga, In, Cr) NCs at the PBE0-D3/def2TZVP level of theory (C/M = Charge/Multiplicity).

(eV)	C/M	Al	C/M	Ga	C/M	In	C/M	Cr
1	2/1	0.37	2/1	0.30	2/1	0.26	2/2	0.06
	**2/3**	**0.00**	**2/3**	**0.00**	**2/3**	**0.00**	2/4	0.0004
	2/5	0.52	2/5	0.47	2/5	0.42	**2/6**	**0.0000**
	--	--	--	--	--	--	2/8	0.63

Table S7. Magnetic moment values (calculated using VASP) and the lowest energy spin multiplicity (calculated using ORCA) for $[Co_5MS_8(PEt_3)_6]^{1+/2+}$ NCs (* = parent cluster).

Dopants	MagMom	ORCA
Co*	1	2
Cr	2/1	5/6
Mn	3	4
Fe	2	3
Ni	0	1
Cu	1	2
Mo	2	3
Pd	0	1
Al	1/2	2/3
Ga	1/2	2/3
In	1/2	2/3

Table S8. SOMO-LUMO Gaps (eV) for $[Co_{6-x}M_xS_8(PEt_3)_6]^{n+}$ (x=1-3, n = 0, 1, 2) NCs at the PBE0-D3/def2TZVP level of theory (* = parent cluster).

(eV)	Charge of NC	x=1	x=2	x=3
Co*	0/1+	3.75/2.91	--	--
Cr	1+/2+	2.78/2.69	2.65/--	2.52/--
Mn	1+	2.83	1.78	2.06
Fe	1+	2.77	--	2.59
Ni	1+	3.04	2.05	2.36
Cu	1+	1.57	--	1.71
Mo	1+	2.09	1.99	2.90
Pd	1+	3.18	1.64	1.79
Al	1+/2+	2.59/2.31	1.69/--	2.01/--
Ga	1+/2+	1.63/2.25	1.76/--	2.18/--
In	1+/2+	1.628/2.23	1.80/--	2.26/--

Table S9. Average bond distances (Å) for $[Co_5MS_8(PEt_3)_6]^{1+}$ NCs in their lowest energy spin multiplicity the PBE0-D3/def2TZVP level of theory (* = parent cluster).

	Co-M	Co-Co	M-S	Co-S	M-P	Co-P
Co*	--	2.756 ± 0.027	--	2.218 ± 0.011	--	2.147 ± 0.029
Cr	2.816 ± 0.010	2.748 ± 0.029	2.323 ± 0.008	2.225 ± 0.018	2.370	2.157 ± 0.039
Mn	2.742 ± 0.001	2.765 ± 0.003	2.287 ± 0.005	2.220 ± 0.015	2.371	2.140 ± 0.004
Fe	2.736 ± 0.009	2.770 ± 0.010	2.258 ± 0.006	2.217 ± 0.011	2.273	2.137 ± 0.004
Ni	2.738 ± 0.003	2.776 ± 0.003	2.218 ± 0.006	2.212 ± 0.007	2.172	2.130 ± 0.003
Cu	2.832 ± 0.003	2.783 ± 0.003	2.269 ± 0.009	2.212 ± 0.006	2.350	2.136 ± 0.002
Mo	2.734 ± 0.061	2.746 ± 0.014	2.373 ± 0.030	2.230 ± 0.031	2.449	2.153 ± 0.009
Pd	2.824 ± 0.002	2.793 ± 0.008	2.333 ± 0.005	2.221 ± 0.012	2.266	2.132 ± 0.002
Al	2.907 ± 0.016	2.746 ± 0.025	2.325 ± 0.024	2.227 ± 0.023	2.416	2.163 ± 0.051
Ga	3.017 ± 0.007	2.803 ± 0.007	2.378 ± 0.020	2.225 ± 0.021	2.383	2.137 ± 0.007
In	3.167 ± 0.023	2.811 ± 0.009	2.531 ± 0.034	2.230 ± 0.027	2.566	2.139 ± 0.009

Undercoordinated (L_5) Nanoclusters:

Table S10. Summary table of the lowest energy spin multiplicity for $[Co_5MS_8(PEt_3)_5]^{n+}$ (n =1, 2) NCs at the PBE0-D3/def2TZVP level of theory (* = parent cluster).

Metal	Charge of NC	Multiplicity
Co*	1+	4
Cr	1+/2+	7/6
Mn	1+	6
Fe	1+	5
Ni	1+	3
Cu	1+	2
Mo	1+	3
Pd	1+	3
Al	1+/2+	2/3
Ga	1+/2+	2/5
In	1+/2+	2/3

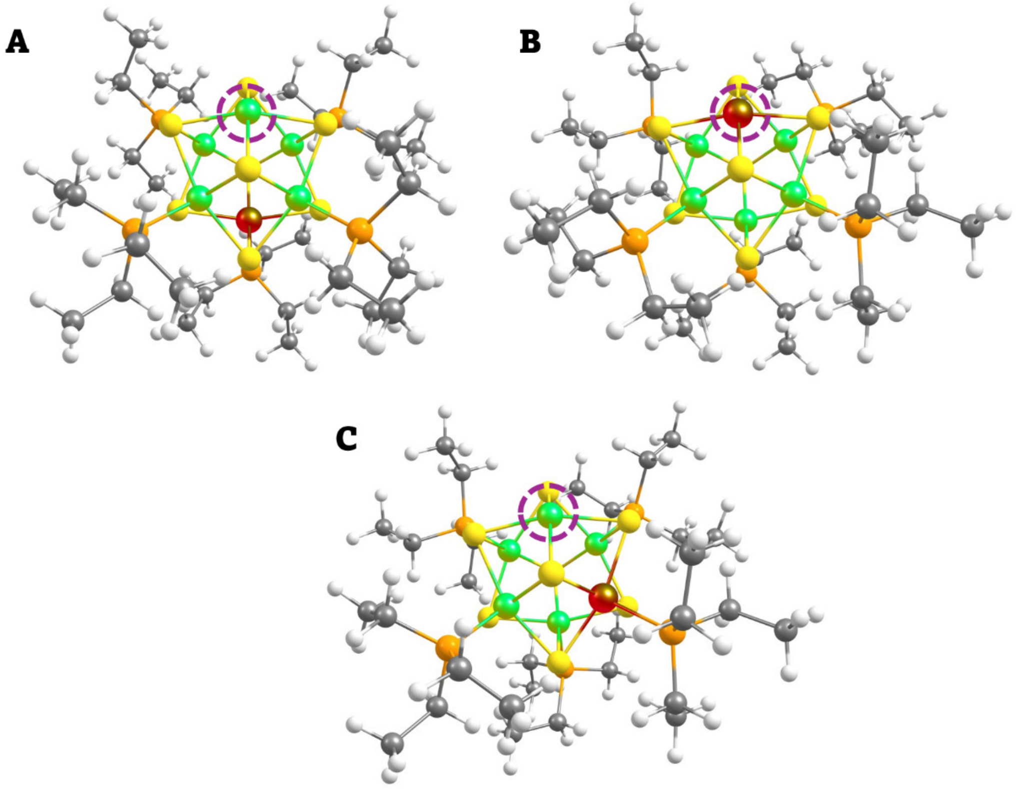

Figure S1. Symmetry isomers of undercoordinated Co_5Fe NCs (A) Co – trans, (B) 'M', M = Cr, Mn, Fe, Ni, Cu, Mo, Pd, Al, Ga, In and (C) Co – cis.

Table S11. Relative energies (eV) at different spin multiplicities and symmetry isomers (Conf) for $[Co_5MS_8(PEt_3)_5]^{1+}$ (M=Co, Cr, Mn) NCs at the PBE0-D3/def2TZVP level of theory (C/M = Charge/Multiplicity).

Co			Cr			Mn		
C/M	Isomer	Relative E	C/M	Isomer	Relative E	C/M	Isomer	Relative E
1/2	M1 - Co	0.01	1/3	Co - cis	0.70	1/2	Mn	1.36
1/4	**M1 - Co**	**0.00**	1/5	Co - cis	0.10	1/4	Co - cis	0.80
	M2 - Co	0.11		Co - trans	0.67		Co - trans	1.50
	M3 - Co	0.11		Cr	0.33	1/6	Co - cis	0.23
	M4 - Co	0.11	1/7	**Co - cis**	**0.00**		Co - trans	0.22
	M5 - Co	0.11		Co - trans	0.17		**Mn**	**0.00**
	M6 - Co	0.11		Cr	0.55	1/8	Co - cis	0.87
1/6	M1 - Co	0.74	1/9	Co - cis	0.64		Co - trans	1.20
							Mn	0.53

Fe			Ni			Cu		
C/M	Isomer	Relative E	C/M	Isomer	Relative E	C/M	Isomer	Relative E
1/1	Co - cis	2.59	1/1	Co - cis	1.14	1/2	Co - cis	0.51
	Fe	2.30		Ni	0.66		Co - trans	0.59
1/3	Co - cis	0.68	1/3	Co - cis (M1)	0.05		**Cu**	**0.00**
	Co - trans	0.74		**Co - trans (M2)**	**0.00**	1/4	Cu	0.60
	Fe	0.29		Co - cis (M3)	0.04	1/6	Cu	1.01
1/5	Co - cis (M1)	0.23		Co - cis (M4)	0.05			
	Co - trans (M2)	0.18		Ni (M5)	0.42			
	Co - cis (M3)	0.22		Co - cis (M6)	0.05			
	Co - cis (M4)	0.23	1/5	Co - cis	0.05			
	**Fe (M5)**	**0.00**		Co - trans	1.01			
	Co - cis (M6)	0.21		Ni	0.90			
1/7	Co - cis	0.71						
	Co - trans	0.18						
	Fe	0.65						

Table S12. Relative energies at different spin multiplicities and symmetry isomers (Conf) for $[Co_5MS_8(PEt_3)_5]^{1+}$ (M=Mo, Pd) NCs at the PBE0-D3/def2TZVP level of theory (C/M = Charge/Multiplicity).

Mo			Pd		
C/M	Isomer	Relative E (eV)	C/M	Isomer	Relative E (eV)
1/1	Co - cis	0.56	1/1	Co - cis	1.14
1/3	**Co - cis**	**0.00**		Co - trans	1.15
	Co - trans	0.31		Pd	0.64
	Mo	0.69	1/3	Co - cis	0.11
1/5	Co - cis	0.09		**Co - trans**	**0.00**
				Pd	0.59
			1/5	Co - cis	0.84
				Co - trans	0.66
				Pd	0.9

Table S13. Relative energies (eV) at different spin multiplicities and symmetry isomers (Conf) for $[Co_5MS_8(PEt_3)_5]^{1+}$ (M=Al, Ga, In) NCs at the PBE0-D3/def2TZVP level of theory (C/M = Charge/Multiplicity).

Al			Ga			In		
C/M	Isomer	Relative E	C/M	Isomer	Relative E	C/M	Isomer	Relative E
1/2	Co - cis	0.16	1/2	Co - cis	0.16	1/2	Co - cis	0.25
	Co - trans	0.27		Co - trans	0.28		Co - trans	0.39
	**Al**	**0.00**		**Ga**	**0.00**		**In**	**0.00**
1/4	Al	0.53	1/4	Ga	0.51	1/4	Co - cis	0.33
1/6	Al	0.97	1/6	Ga	1.17		Co - trans	0.51
							In	0.33
						1/6	Co - cis	0.85

Table S14. Relative energies at different spin multiplicities and symmetry isomers (Conf) for $[Co_5MS_8(PEt_3)_5]^{2+}$ (M=Al, Ga, In, Cr) NCs at the PBE0-D3/def2TZVP level of theory (C/M = Charge/Multiplicity).

Al			Ga		
C/M	Isomer	Relative E (eV)	C/M	Isomer	Relative E (eV)
2/1	Al	0.69	2/1	Co - cis	1.33
2/3	Co - cis (M1)	0.06	2/3	Co - cis (M1)	0.05
	Co - trans (M2)	0.24		Co - trans (M2)	0.30
	Co - cis (M3)	0.10		Co - cis (M3)	0.03
	Co - cis (M4)	SCF		Co - cis (M4)	0.13
	Al (M5)	0.02		Ga (M5)	0.11
	**Co - cis (M6)**	**0.00**		Co - cis (M6)	2.75E-05
2/5	Al	0.73	2/5	**Co - cis**	**0.00**
				Co - trans	0.19
					0.69
			2/7	Co - cis	0.35

In			Cr		
C/M	Isomer	Relative E (eV)	C/M	Isomer	Relative E (eV)
2/1	Co - cis	1.41	2/2	Co - cis	0.67
2/3	Co - cis (M1)	0.07	2/4	Co - cis	0.25
	Co - trans (M2)	0.19	2/6	Co - cis (M1)	0.12
	Co - cis (M3)	0.03		Co - trans (M2)	0.20
	Co - cis (M4)	SCF		Co - cis (M3)	0.28
	In (M5)	0.09		Co - cis (M4)	0.16
	**Co - cis (M6)**	**0.00**		Cr (M5)	0.38
2/5	Co - cis	0.02		**Co - cis (M6)**	**0.00**
	Co - trans	0.45	2/8	Co - cis	0.10
	In	0.75		Co - trans	0.23
2/7	Co - cis	0.37		Cr	0.56
			2/10	Co - cis	0.46
				Co - trans	0.50
				Cr	1.15

Table S15. Magnetic moment values (calculated using VASP) and the lowest energy spin multiplicity (calculated using ORCA) for $[Co_5MS_8(PEt_3)_5]^{1+/2+}$ NCs (* = parent cluster).

	Dopants	MagMom	ORCA
L5	Co*	3	4
L5	Cr	4/5	7/6
L5	Mn	5	6
L5	Fe	4	5
L5	Ni	2	3
L5	Cu	1	2
L5	Mo	0	3
L5	Pd	2	3
L5	Al	1/2	2/3
L5	Ga	1/4	2/5
L5	In	1/4	2/3

Table S16. SOMO-LUMO Gaps (eV) for $[Co_5MS_8(PEt_3)_6]^{n+}$ (n = 0, 1, 2) NCs at the PBE0-D3/def2TZVP level of theory (* = parent cluster).

(eV)	Charge of NC	Gap (eV)
Co*	0/1+	2.45/2.70*
Cr	1+/2+	2.82/2.47
Mn	1+	2.61
Fe	1+	2.21
Ni	1+	2.93
Cu	1+	1.81
Mo	1+	1.99
Pd	1+	2.99
Al	1+/2+	2.78/1.98
Ga	1+/2+	2.63/1.92
In	1+/2+	2.54/1.85

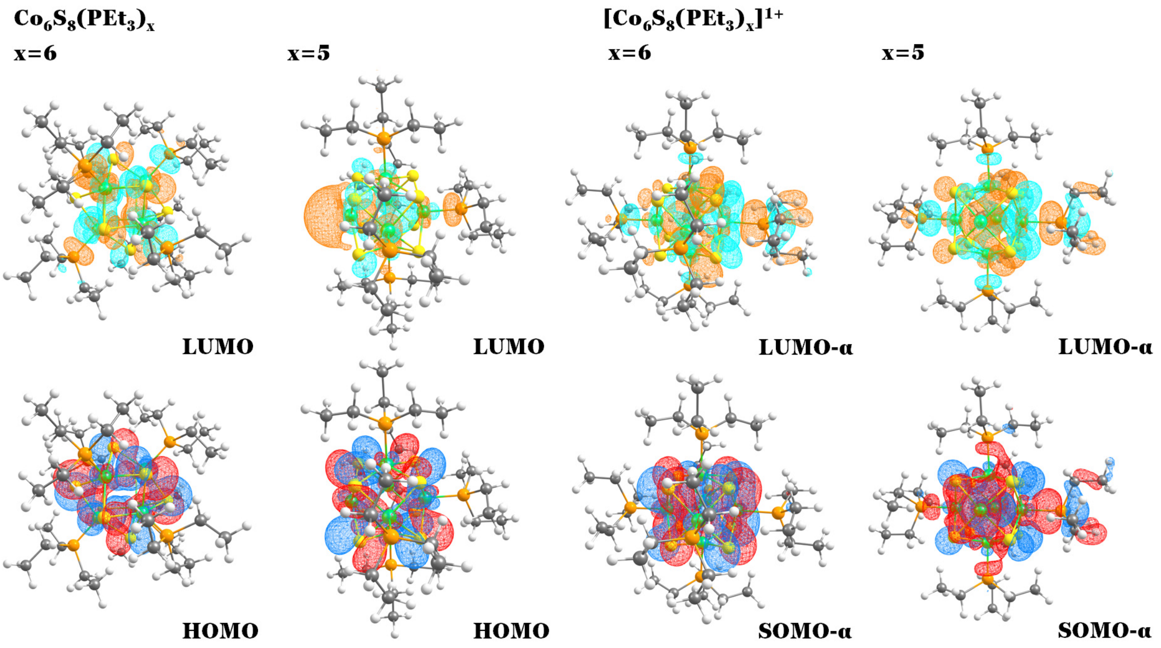

Figure S2. SOMO/LUMO molecular orbitals of $[Co_6S_8(PEt_3)_x]^{0/1+}$ at PBE0-D3/def2TZVP(opt)/CEP-31G(freq) level of theory.

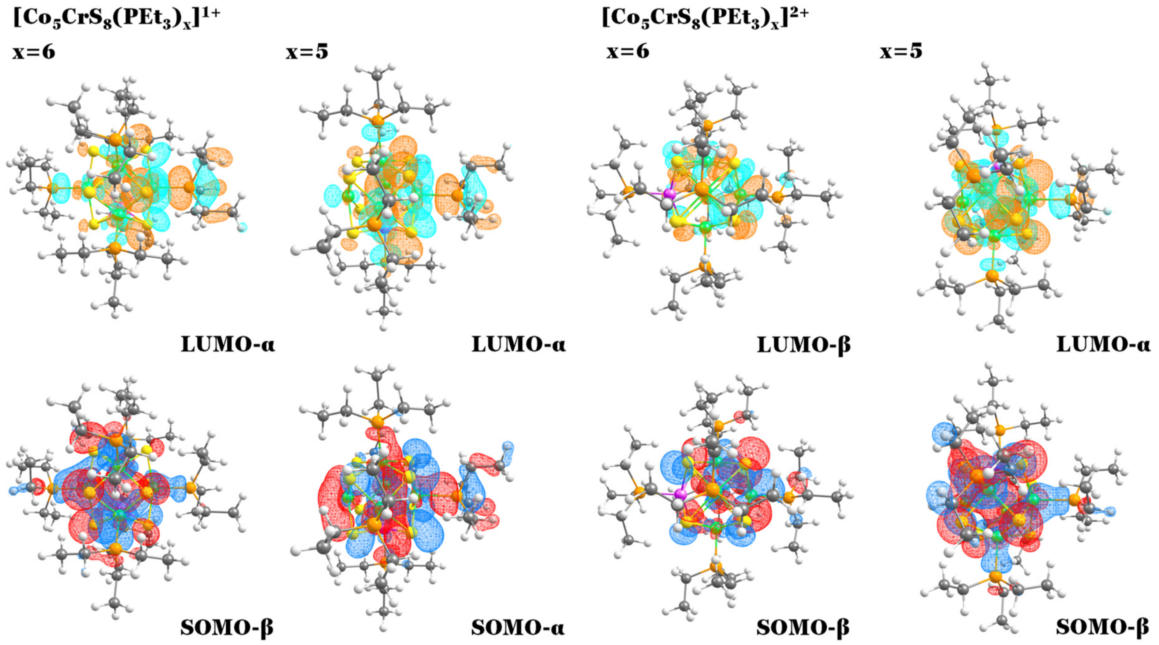

Figure S3. SOMO/LUMO molecular orbitals of $[Co_5CrS_8(PEt_3)_x]^{1+/2+}$ at PBE0-D3/def2TZVP(opt)/CEP-31G(freq) level of theory.

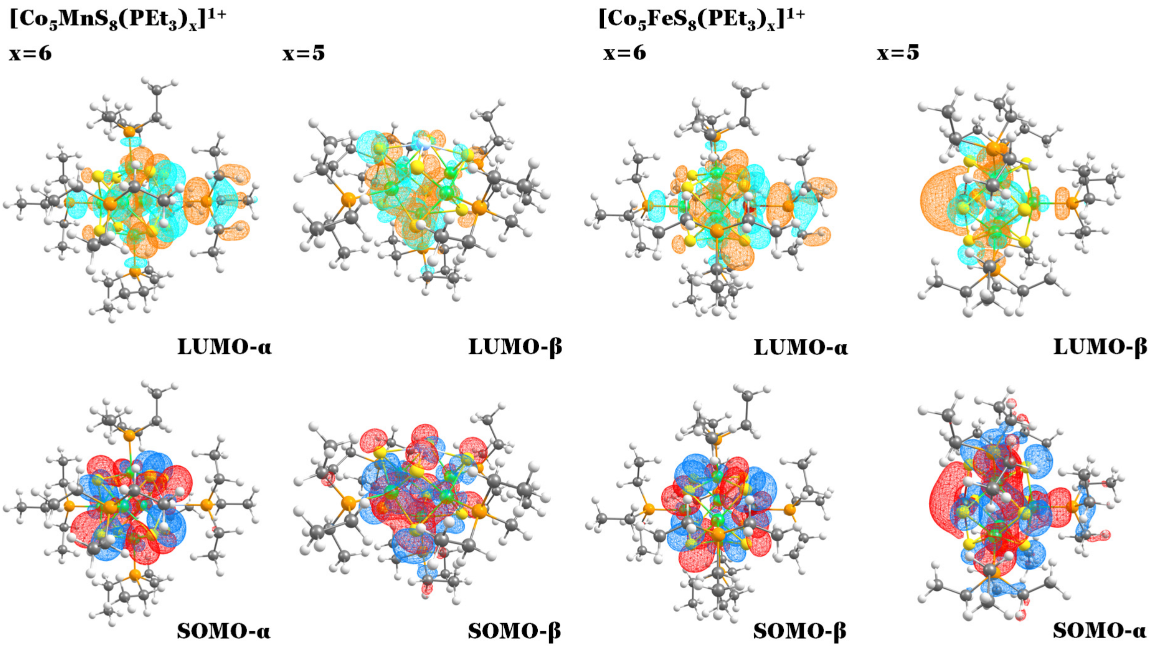

Figure S4. SOMO/LUMO molecular orbitals of $[Co_5MS_8(PEt_3)_x]^{1+}$ (M=Mn, Fe) at PBE0-D3/def2TZVP(opt)/CEP-31G(freq) level of theory.

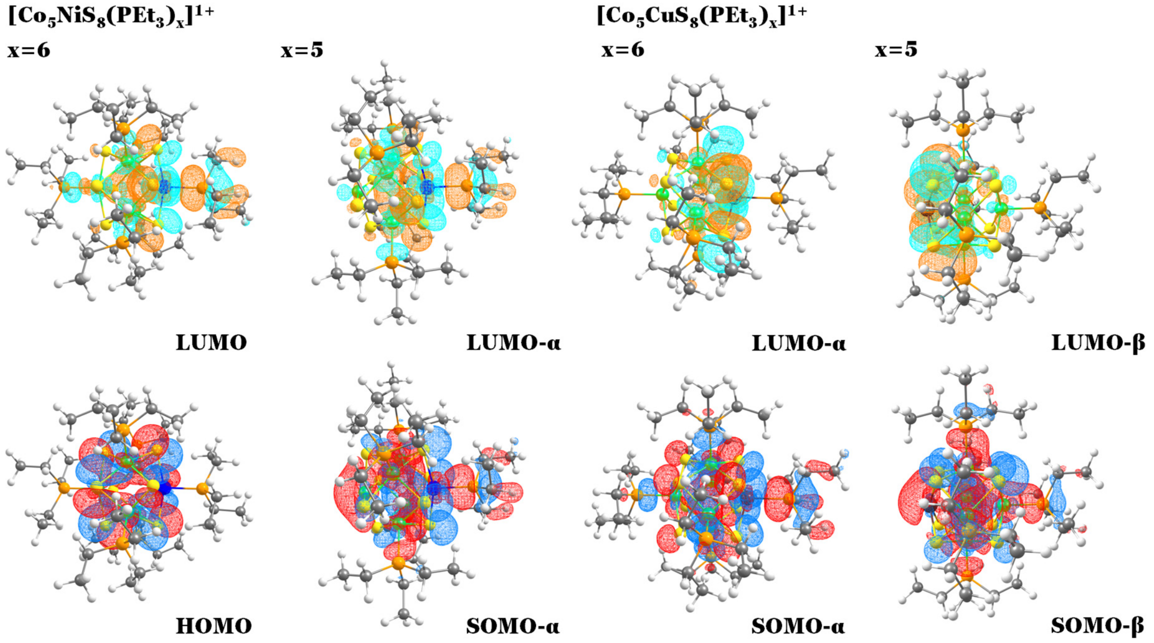

Figure S5. SOMO/LUMO molecular orbitals of $[Co_5MS_8(PEt_3)_x]^{1+}$ (M=Ni, Cu) at PBE0-D3/def2TZVP(opt)/CEP-31G(freq) level of theory.

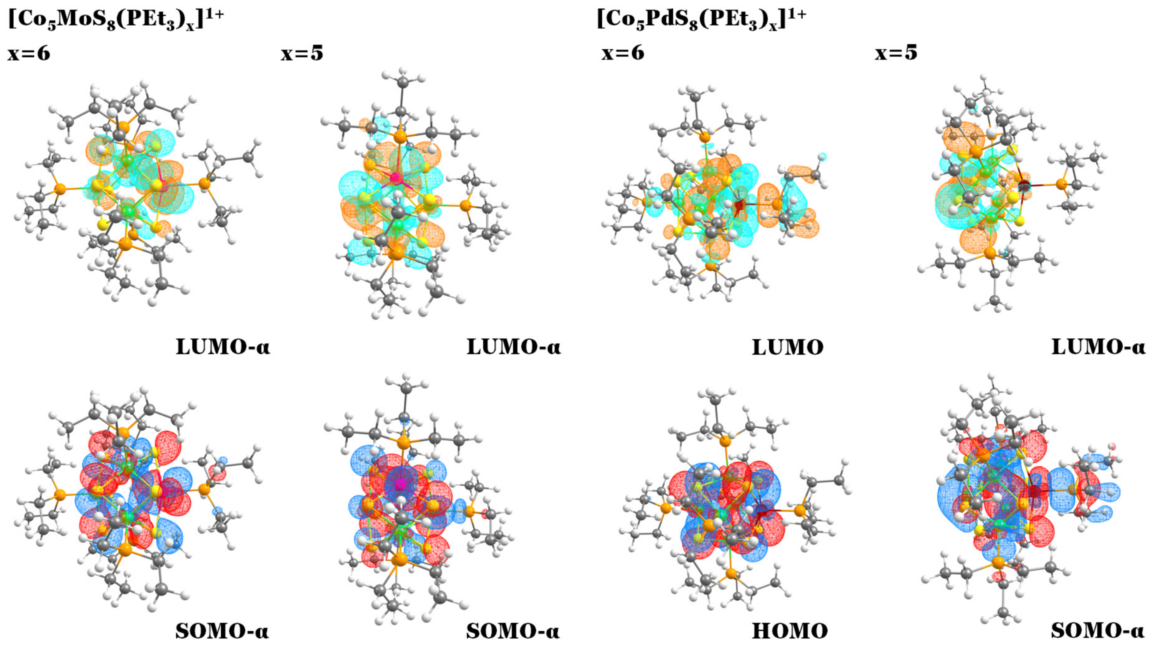

Figure S6. SOMO/LUMO molecular orbitals of $[Co_5MS_8(PEt_3)_x]^{1+}$ (M=Mo, Pd) at PBE0-D3/def2TZVP(opt)/CEP-31G(freq) level of theory.

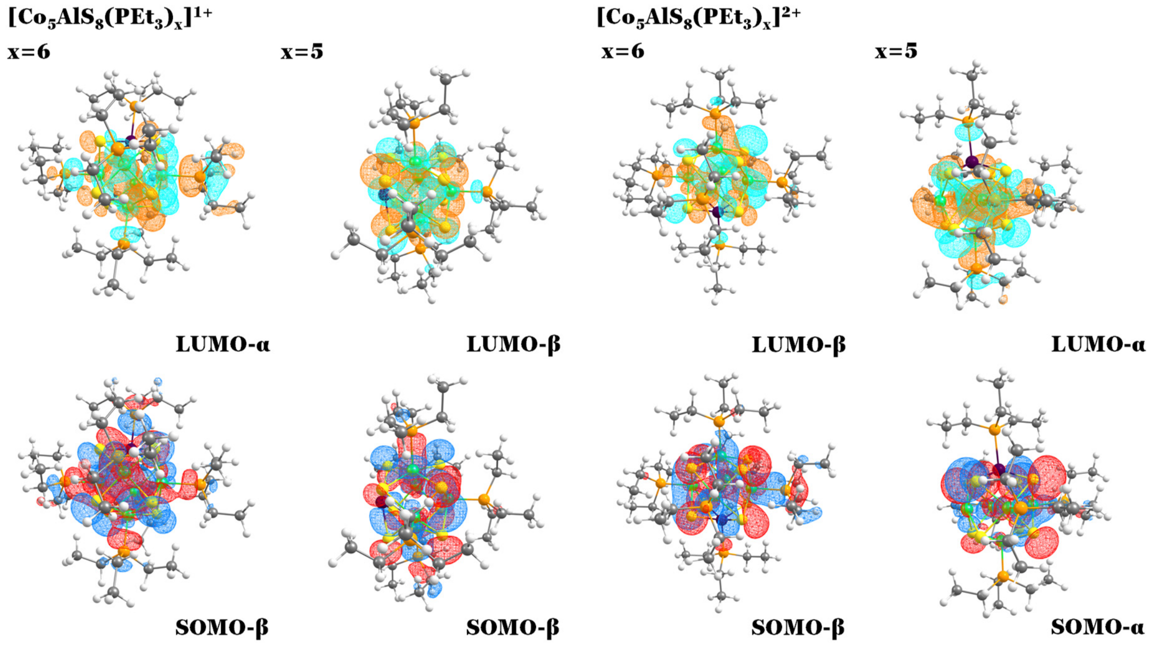

Figure S7. SOMO/LUMO molecular orbitals of $[Co_5AlS_8(PEt_3)_x]^{1+/2+}$ at PBE0-D3/def2TZVP(opt)/CEP-31G(freq) level of theory.

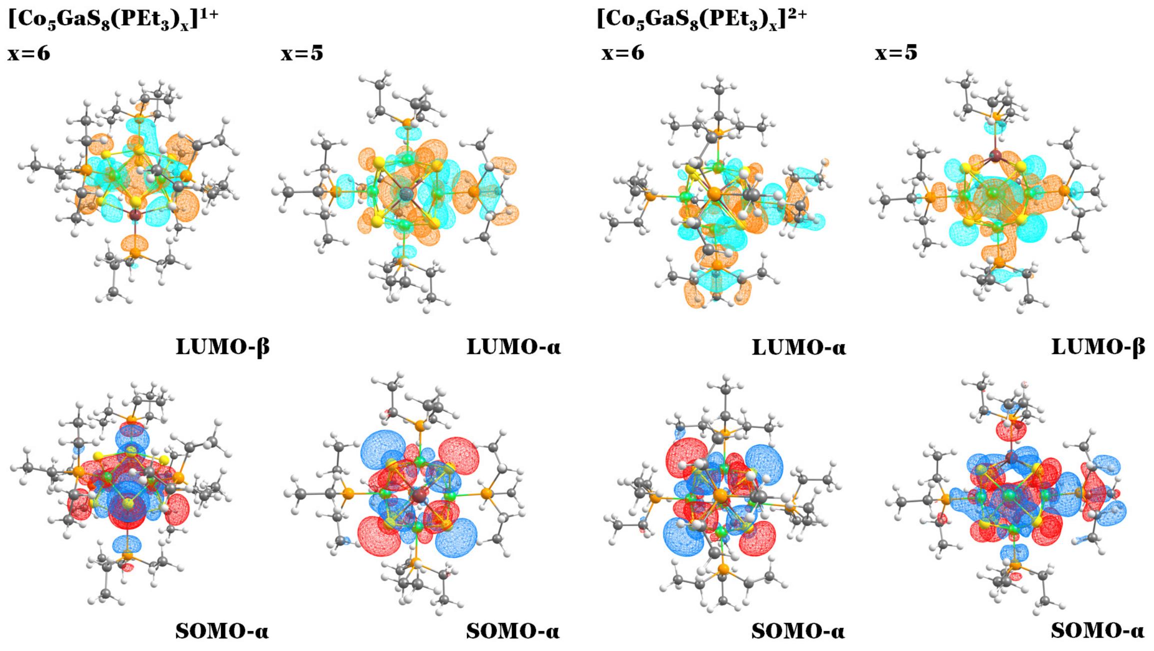

Figure S8. SOMO/LUMO molecular orbitals of $[Co_5GaS_8(PEt_3)_x]^{1+/2+}$ at PBE0-D3/def2TZVP(opt)/CEP-31G(freq) level of theory.

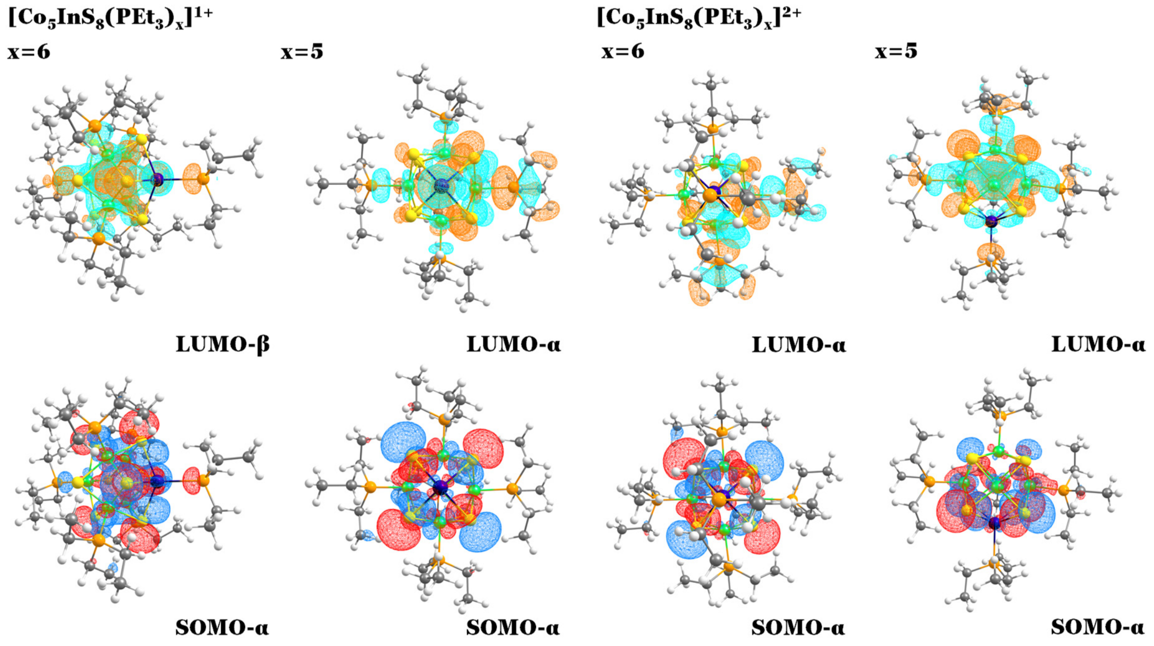

Figure S9. SOMO/LUMO molecular orbitals of $[Co_5InS_8(PEt_3)_x]^{1+/2+}$ at PBE0-D3/def2TZVP(opt)/CEP-31G(freq) level of theory.

Ligand Binding Calculations - L_5 and L_6 Nanoclusters:

All reaction energies were calculated with total energy from gas-phase calculations (ORCA), adding a zero-point energy correction (Gaussian), and adding a Gibbs thermodynamic correction at 298K (Gaussian). Table S17 shows an exact calculation of obtaining E, EZPE, and G for each compound. Reaction S1 shows the reaction scheme, equations S1-S3 show the mathematical definitions.

Reaction S1. $[Co_5MS_8(PEt_3)_6]^{1+} \rightarrow [Co_5MS_8(PEt_3)_5]^{1+} + (PEt_3)_5$

Equation S1. $\Delta E = [\text{Energy}\ ([Co_5MS_8(PEt_3)_5]^{1+} + \text{Energy}\ (PEt_3)_5] - \text{Energy}\ ([Co_5MS_8(PEt_3)_6]^{1+})$

Equation S2 – Zero-point Energy Correction (ORCA + Gaussian Correction at 298.15K)
$\Delta E_{ZPE} = [E_{ZPE}([Co_5MS_8(PEt_3)_5]^{1+}) + E_{ZPE}((PEt_3)_5)] - [E_{ZPE}([Co_5MS_8(PEt_3)_6]^{1+})]$

Equation S3 – Gibbs-Free Energy Correction (ORCA + Gaussian Correction at 298.15K)
$\Delta G_{298.15} = [\text{Energy} + \text{Gibbs correction}\ ([Co_5MS_8(PEt_3)_5]^{1+}) + \text{Energy} + \text{Gibbs Correction}\ ((PEt_3)_5)] - \text{Energy} + \text{Gibbs Correction}\ ([Co_5MS_8(PEt_3)_6]^{1+})$

Table S17. Example of the Zero-point energy and Gibbs free energy corrections for the calculation of ligand binding in $Co_{6-x}Fe_x$ (x = 1, 3) NCs at the PBE0-D3/def2TZVP (ORCA – geometry optimizations) and PBE0-D3/CEP-31G (Gaussian - single point calculations) level of theory. All values are shown in Hartree.

(PEt3)	Total Energy (ORCA)	-578.7160561			
(0/1)	ZPE Correction (Gaussian)	0.203149			
	G Correction (Gaussian)	0.170378			
	EZPE	-578.5129071			
	G	-578.5456781			
**(PEt3)6 NCs**			**(PEt3)5 NCs**		
Co6	Total Energy (ORCA)	-14953.00157	Co6	Total Energy (ORCA)	-14374.20305
(1/2)	ZPE Correction (Gaussian)	1.263446	(1/4)	ZPE Correction (Gaussian)	1.057345
	G Correction (Gaussian)	1.163217		G Correction (Gaussian)	0.966758
	EZPE	-14951.73812		EZPE	-14373.1457
	G	-14951.83835		G	-14373.23629
Co5Fe	Total Energy (ORCA)	-14833.95307	Co5Fe	Total Energy (ORCA)	-14255.16344
(1/3)	ZPE Correction (Gaussian)	1.263307	(1/5)	ZPE Correction (Gaussian)	1.057452
	G Correction (Gaussian)	1.16344		G Correction (Gaussian)	0.966854
	EZPE	-14832.68977		EZPE	-14254.10599
	G	-14832.78963		G	-14254.19659

NBO Analysis of L_5 and L_6 Nanoclusters:

Table S18. POS value at '0.65' value threshold for $[Co_5MS_8(PEt_3)_6]^{1+}$ NCs (* = parent cluster) at the lowest spin multiplicity.

L6	M1	M2	M3	M4	M5	M6
Co*	1	1	0	2	2	1
Cr	1	2	2	1	3	3
Mn	2	2	2	2	2	2
Fe	2	2	2	2	2	2
Ni	1	1	1	1	0	1
Cu	2	2	2	2	1	2
Mo	2	1	2	2	4	2
Pd	1	1	1	1	0	1
Al	2	2	2	2	--	2
Ga	2	2	2	2	--	2
In	2	2	2	2	--	2

Table S19. POS value at '0.65' value threshold for $[Co_5MS_8(PEt_3)_5]^{1+}$ NCs (* = parent cluster) at the lowest spin multiplicity.

L5	M1	M2	M3	M4	M5	M6
Co	2	3	2	2	2	2
Cr	2	3	2	2	3	2
Mn	2	1	1	1	2	2
Fe	2	2	2	2	2	2
Ni	2	2	2	2	0	2
Cu	2	2	2	2	1	2
Mo	1	2	1	2	5	1
Pd	2	2	2	2	0	2
Al	1	2	1	1	--	1
Ga	2	2	2	2	--	2
In	2	2	2	2	--	2

Table S20. Total alpha and beta d-orbital occupancy for each metal center in $[Co_5MS_8(PEt_3)_6]^{1+}$ (M=Co, Cr, Mn) NCs at the lowest spin multiplicity (pink = dopant).

**Co***	M1	M2	M3	M4	M5	M6	d-occ in NC
Total Spin Up	4.23	4.22	4.33	4.21	4.19	4.25	
Total Spin Down	4.24	4.23	4.12	4.24	4.27	4.23	
Occ. On Metal	8.47	8.45	8.45	8.45	8.46	8.48	50.76
**Cr**							
Total Spin Up	4.24	4.21	4.23	4.68	4.12	4.23	
Total Spin Down	4.22	4.26	4.22	3.48	1.31	4.19	
Occ. On Metal	8.46	8.47	8.45	8.16	5.43	8.42	47.39
**Mn**							
Total Spin Up	4.21	4.20	4.21	4.21	4.61	4.21	
Total Spin Down	4.24	4.26	4.24	4.24	1.54	4.24	
Occ. On Metal	8.45	8.46	8.45	8.45	6.15	8.45	48.41

Table S21. Total alpha and beta d-orbital occupancy for each metal center in $[Co_5MS_8(PEt_3)_6]^{1+}$ (M=Fe, Ni, Cu) NCs at the lowest spin multiplicity (pink = dopant).

**Fe**	M1	M2	M3	M4	M5	M6	d-occ in NC
Total Spin Up	4.20	4.20	4.22	4.20	4.68	4.21	
Total Spin Down	4.25	4.25	4.24	4.24	2.47	4.25	
Occ. On Metal	8.45	8.45	8.46	8.44	7.15	8.46	49.41
**Ni**							
Total Spin Up	4.23	4.23	4.24	4.23	4.54	4.24	
Total Spin Down	4.23	4.23	4.24	4.23	4.54	4.24	
Occ. On Metal	8.46	8.46	8.48	8.46	9.08	8.48	51.42
**Cu**							
Total Spin Up	4.28	4.28	4.29	4.30	4.87	4.29	
Total Spin Down	4.19	4.16	4.18	4.18	4.77	4.19	
Occ. On Metal	8.47	8.44	8.47	8.48	9.64	8.48	51.98

Table S22. Total alpha and beta d-orbital occupancy for each metal center in $[Co_5MS_8(PEt_3)_6]^{1+}$ (M=Mo, Pd) NCs at the lowest spin multiplicity (pink = dopant).

**Mo**	M1	M2	M3	M4	M5	M6	d-occ in NC
Total Spin Up	4.28	4.22	4.30	4.30	3.52	4.19	
Total Spin Down	4.18	4.26	4.18	4.17	2.04	4.31	
Occ. On Metal	8.46	8.48	8.48	8.47	5.56	8.50	47.95
**Pd**							
Total Spin Up	4.22	4.23	4.23	4.22	4.58	4.23	
Total Spin Down	4.22	4.23	4.23	4.22	4.58	4.23	
Occ. On Metal	8.44	8.46	8.46	8.44	9.16	8.46	51.42

Table S23. Total alpha and beta d-orbital occupancy for each metal center in $[Co_5MS_8(PEt_3)_6]^{1+}$ (M=Al, Ga, In) NCs at the lowest spin multiplicity.

**Al**	M1	M2	M3	M4	M5	M6	d-occ in NC
Total Spin Up	4.19	4.20	4.16	4.17	--	4.72	
Total Spin Down	4.27	4.26	4.29	4.30	--	3.37	
Occ. On Metal	8.46	8.46	8.45	8.47	--	8.09	41.93
**Ga**							
Total Spin Up	4.32	4.31	4.30	4.31	--	4.32	
Total Spin Down	4.10	4.13	4.12	4.13	--	4.08	
Occ. On Metal	8.42	8.44	8.42	8.44	--	8.40	42.12
**In**							
Total Spin Up	4.32	4.31	4.31	4.31	--	4.32	
Total Spin Down	4.09	4.13	4.11	4.11	--	4.07	
Occ. On Metal	8.41	8.44	8.42	8.42	--	8.39	42.08

Table S24. Total alpha and beta d-orbital occupancy for each metal center in $[Co_5MS_8(PEt_3)_5]^{1+}$ (M=Co, Cr, Mn) NCs at the lowest spin multiplicity (pink = dopant).

**Co***	M1	M2	M3	M4	M5	M6	d-occ in NC
Total Spin Up	4.80	4.24	4.18	4.18	4.74	4.23	
Total Spin Down	2.85	4.21	4.28	4.29	3.32	4.24	
Occ. On Metal	7.65	8.45	8.46	8.47	8.06	8.47	49.56
**Cr**							
Total Spin Up	4.81	4.22	4.17	4.17	4.11	4.74	
Total Spin Down	2.84	4.21	4.29	4.31	1.29	3.29	
Occ. On Metal	7.65	8.43	8.46	8.48	5.40	8.03	46.45
**Mn**							
Total Spin Up	4.21	4.53	4.23	4.55	4.68	4.18	
Total Spin Down	4.25	3.80	4.24	3.78	0.96	4.28	
Occ. On Metal	8.46	8.33	8.47	8.33	5.64	8.46	47.69

Table S25. Total alpha and beta d-orbital occupancy for each metal center in $[Co_5MS_8(PEt_3)_5]^{1+}$ (M=Fe, Ni, Cu) NCs at the lowest spin multiplicity (pink = dopant).

**Fe**	M1	M2	M3	M4	M5	M6	d-occ in NC
Total Spin Up	4.32	4.32	4.32	4.31	4.79	4.31	
Total Spin Down	4.14	4.12	4.14	4.14	1.81	4.15	
Occ. On Metal	8.46	8.44	8.46	8.45	6.60	8.46	48.87
**Ni**							
Total Spin Up	4.25	4.80	4.19	4.19	4.63	4.25	
Total Spin Down	4.21	2.81	4.27	4.27	4.44	4.21	
Occ. On Metal	8.46	7.61	8.46	8.46	9.07	8.46	50.52
**Cu**							
Total Spin Up	4.15	4.19	4.18	4.72	4.82	4.17	
Total Spin Down	4.32	4.25	4.28	3.39	4.81	4.30	
Occ. On Metal	8.47	8.44	8.46	8.11	9.63	8.47	51.58

Table S26. Total alpha and beta d-orbital occupancy for each metal center in $[Co_5MS_8(PEt_3)_5]^{1+}$ (M=Mo, Pd) NCs at the lowest spin multiplicity (pink = dopant).

**Mo**	M1	M2	M3	M4	M5	M6	d-occ in NC
Total Spin Up	4.62	4.22	4.05	4.29	3.16	4.40	
Total Spin Down	3.40	4.26	4.42	4.18	2.51	4.08	
Occ. On Metal	8.02	8.48	8.47	8.47	5.67	8.48	47.59
**Pd**							
Total Spin Up	4.26	4.80	4.21	4.21	4.59	4.26	
Total Spin Down	4.19	2.82	4.24	4.24	4.57	4.19	
Occ. On Metal	8.45	7.62	8.45	8.45	9.16	8.45	50.58

Table S27. Total alpha and beta d-orbital occupancy for each metal center in $[Co_5MS_8(PEt_3)_5]^{1+}$ (M=Al, Ga, In) NCs at the lowest spin multiplicity (pink = dopant).

**Al**	M1	M2	M3	M4	M5	M6	d-occ in NC
Total Spin Up	4.45	4.27	4.24	4.25	--	4.36	
Total Spin Down	3.94	4.20	4.25	4.24	--	4.03	
Occ. On Metal	8.39	8.47	8.49	8.49	--	8.39	42.23
**Ga**							
Total Spin Up	4.21	4.21	4.19	4.19	--	4.71	
Total Spin Down	4.27	4.27	4.29	4.29	--	3.41	
Occ. On Metal	8.48	8.48	8.48	8.48	--	8.12	42.04
**In**							
Total Spin Up	4.19	4.20	4.16	4.17	--	4.72	
Total Spin Down	4.27	4.27	4.29	4.30	--	3.37	
Occ. On Metal	8.46	8.47	8.45	8.47	--	8.09	41.94

Hydrogen Evolution Reaction (HER) on Undercoordinated (L_5) Nanoclusters:

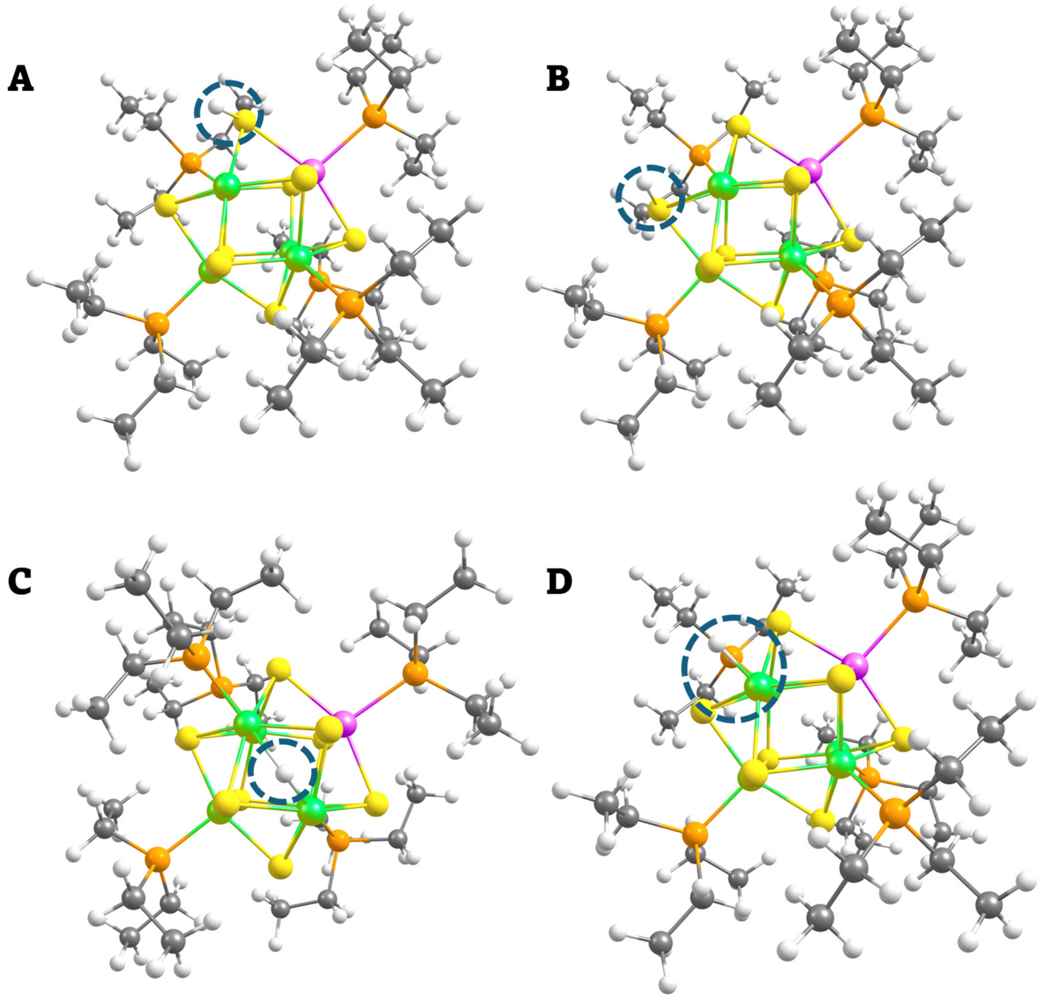

Figure S10. Initial starting geometries for *H species in each NC. (A) S1 – Sulfur next to metal dopant, (B) S2 – Sulfur next to cobalt across from metal dopant, (C) Bridged site between two metal atoms, (D) Directly on the metal atom.

Table S28. Relative energies (eV) at different spin multiplicities and symmetry isomers (Conf) for *H adsorption or $[Co_5MS_8(PEt_3)_5H]^{1+}$ (M=Co, Cr, Mn, Fe, Ni, Cu) NCs at the PBE0-D3/def2TZVP level of theory (C/M = Charge/Multiplicity).

Co			Cr			Mn		
C/M	Isomer	Relative E	C/M	Isomer	Relative E	C/M	Isomer	Relative E
1/1	S	0.88	1/4	S2	0.53	1/3	S	1.41
1/3	**S**	**0.00**	1/6	**S1**	**0.00**		M	0.88
1/5	S	0.31		S2	0.02		Br	1.17
				M	0.85	1/5	S	0.04
				Br	0.05		M	0.91
			1/8	S2	0.46	1/7	**S**	**0.00**
							M	1.53
Fe			Ni			Cu		
C/M	Isomer	Relative E (eV)	C/M	Isomer	Relative E (eV)	C/M	Isomer	Relative E (eV)
1/2	S	0.69	1/2	S	0.14	1/1	**S**	**0.00**
1/4	S	0.12		M	0.25	1/3	S	0.26
	M	0.60	1/4	**S**	**0.00**		M	0.22
1/6	**S**	**0.00**		M	1.37			

Table S29. Relative energies (eV) at different spin multiplicities and symmetry isomers (Conf) for *H adsorption or $[Co_5MS_8(PEt_3)_5H]^{1+}$ (M=Mo, Pd) NCs at the PBE0-D3/def2TZVP level of theory (C/M = Charge/Multiplicity).

Mo			Pd		
C/M	Isomer	Relative E	C/M	Isomer	Relative E
1/2	S1	0.63	1/2	S	0.12
	M	0.39		M	0.23
	Br	0.17	1/4	**S**	**0.00**
1/4	S1	0.10		M	0.92
	**S2**	**0.00**			
1/6	S1	0.12			

Table S30. Relative energies (eV) at different spin multiplicities and symmetry isomers (Conf) for *H adsorption or $[Co_5MS_8(PEt_3)_5H]^{1+}$ (M=Al, Ga, In) NCs at the PBE0-D3/def2TZVP level of theory (C/M = Charge/Multiplicity).

Al			Ga			In		
C/M	Isomer	Relative E	C/M	Isomer	Relative E	C/M	Isomer	Relative E
1/1	**S**	**0.00**	1/1	**S**	**0.00**	**1/1**	**S**	**0.00**
	Br	0.34		M	1.86	1/3	S	0.52
1/3	S	0.62		Br	0.66		M	0.78
			1/3	S	0.61		Br	0.50

Table S31. Relative energies (eV) at different spin multiplicities and symmetry isomers (Conf) for *H adsorption or $[Co_5MS_8(PEt_3)_5H]^{2+}$ (M=Al, Ga, In) NCs at the PBE0-D3/def2TZVP level of theory (C/M = Charge/Multiplicity).

Al			Ga		
C/M	Isomer	Relative E (eV)	C/M	Isomer	Relative E (eV)
**2/2**	**S1**	**0.00**	**2/2**	**S1**	**0.00**
	S2	3.14E-04		S2	0.93
	M	0.73	2/4	S1	0.14
2/4	S1	0.03		S2	0.96
	S2	0.39	2/6	S1	0.41
	M	0.70			
**In**			**Cr**		
C/M	Isomer	Relative E (eV)	C/M	Isomer	Relative E (eV)
**2/2**	**S1**	**0.00**	2/5	S1	0.16
	S2	0.02		S2	0.44
	M	0.84		M	1.11
2/4	S1	0.04	**2/7**	**S1**	**0.00**
	S2	0.04		S2	0.45
	M	0.78		M	1.12

Table S32. Reaction energies for hydrogen adsorption following reaction 1, n=1 (C/M = Charge/multiplicity, ZPE = Zero-point energy).

	C/M – L5	C/M – H*	Isomer	ΔE (eV)	ΔE_{ZPE} (eV)	$\Delta G_{298.15}$ (eV)
Co	1/4	1/3	S	-0.30	-0.21	-0.05
Cr	1/7	1/6	S1	-0.21	-0.16	0.05
Mn	1/6	1/7	S	-0.02	0.00	0.16
Fe	1/5	1/6	S	-0.05	0.03	0.22
Ni	1/3	1/4	S	0.30	0.37	0.52
Cu	1/2	1/1	S	-0.44	-0.35	-0.20
Mo	1/3	1/4	S2	0.02	0.07	0.15
Pd	1/3	1/4	S	0.31	0.37	0.45
Al	1/2	1/1	S	-0.59	-0.48	-0.31
Ga	1/2	1/1	S	-0.90	-0.79	-0.72
In	1/2	1/1	S	-0.68	-0.57	-0.43

CO_2 Reduction on Undercoordinated (L_5) Nanoclusters:

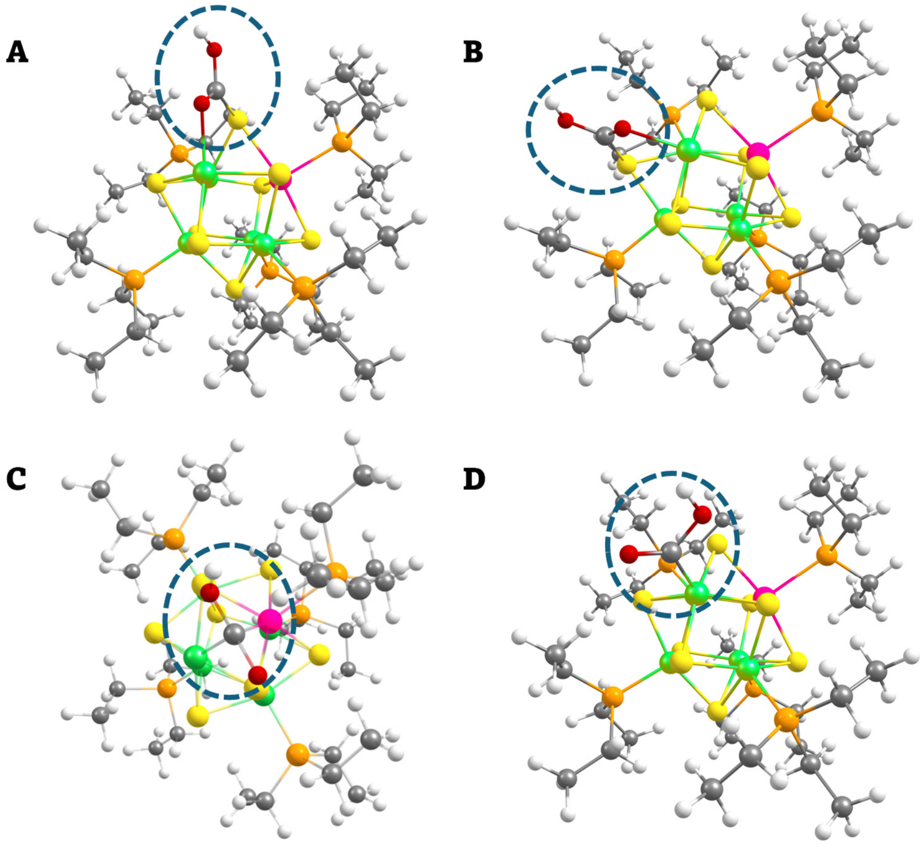

Figure S11. Initial starting geometries for *COOH species (circled) in each NC. (A) S1 – Sulfur next to metal dopant, (B) S2 – Sulfur next to cobalt across from metal dopant, (C) Bridged site between two metal atoms, (D) Directly on the metal atom.

Table S33. Relative energies (eV) at different spin multiplicities and symmetry isomers (Conf) for the first intermediate of CO_2 reduction (*COOH or HCOO*) with M=Co, Cr, Mn NCs at the PBE0-D3/def2TZVP level of theory (C/M = Charge/Multiplicity).

Co	COOH	
C/M	Isomer	Relative E
1/1	S	0.71
	M	1.80
	Br	1.01
**1/3**	**S**	**0.00**
1/5	S	0.41
	HCOO	
1/1	S	1.32
	M	1.35
	Br	1.30

Cr	COOH	
C/M	Isomer	Relative E
1/4	S1	0.59
**1/6**	**S1**	**0.00**
	S2	0.05
	M	0.99
	Br	0.27
1/8	S	0.48
	HCOO	
1/6	S	1.61
	M	1.32
	Br	1.70

Mn	COOH	
C/M	Isomer	Relative E
1/3	S	1.40
	M	1.37
	Br	2.46
**1/5**	**S**	**0.00**
	M	1.39
1/7	S	0.40
	M	2.01
	HCOO	
1/3	S	2.16
	M	1.18
	Br	0.85
1/5	S	0.78
	M	0.91
1/7	S	0.90
	M	1.49

Table S34. Relative energies (eV) at different spin multiplicities and symmetry isomers (Conf) for the first intermediate of CO_2 reduction (*COOH or HCOO*) with M=Fe, Ni, Cu NCs at the PBE0-D3/def2TZVP level of theory (C/M = Charge/Multiplicity).

Fe	COOH	
C/M	Isomer	Relative E
1/2	S	0.90
1/4	S	0.14
	M	1.21
	Br	0.89
**1/6**	**S**	**0.00**
	HCOO	
1/4	S	0.81
	M	0.94
	Br	2.32

Ni	COOH	
C/M	Isomer	Relative E
1/2	S	0.28
	M	0.47
**1/4**	**S**	**0.00**
	M	1.23
	HCOO	
1/2	S	1.18
	M	0.63
1/4	S	0.71
	M	1.23

Cu	COOH	
C/M	Isomer	Relative E
**1/1**	**S**	**0.00**
1/3	S	0.34
	M	1.60
	Br	0.65
	HCOO	
1/3	S	0.34
	M	1.60
	Br	0.65

Table S35. Relative energies (eV) at different spin multiplicities and symmetry isomers (Conf) for the first intermediate of CO_2 reduction (*COOH or HCOO*) with M=Mo, Pd NCs at the PBE0-D3/def2TZVP level of theory (C/M = Charge/Multiplicity).

Mo	COOH		Pd	COOH	
C/M	Isomer	Relative E (eV)	C/M	Isomer	Relative E (eV)
**1/2**	**S1**	**0.00**	1/2	S	0.27
	S2	0.004		M	0.43
	M	0.42	**1/4**	**S**	**0.00**
	Br	0.34		M	1.39
1/4	S	0.25			
HCOO			HCOO		
1/2	S1	1.10	1/2	S	0.61
	M	0.58		M	0.60
	Br	0.26	1/4	S	0.69
				M	0.77

Table S36. Relative energies (eV) at different spin multiplicities and symmetry isomers (Conf) for the first intermediate of CO_2 reduction (*COOH or HCOO*) with M=Al, Ga, In NCs at the PBE0-D3/def2TZVP level of theory (C/M = Charge/Multiplicity).

Al	COOH		Ga	COOH		In	COOH	
C/M	Isomer	Relative E	C/M	Isomer	Relative E	C/M	Isomer	Relative E
**1/1**	**S**	**0.00**	**1/1**	**S**	**0.00**	**1/1**	**S**	**0.00**
	M	2.19		M	1.79		M	1.54
	Br	0.58		Br	0.79	1/3	S	0.63
1/3	S	0.42	1/3	S	0.88		M	1.36
HCOO			HCOO			HCOO		
1/1	S	0.09	1/1	S	0.17	1/1	S	1.36
	M	0.19		M	SCF		M	0.87
	Br	1.00		Br	1.15	1/3	S	SCF
							M	0.44

Table S37. Relative energies (eV) at different spin multiplicities and symmetry isomers (Conf) for the first intermediate of CO_2 reduction (*COOH or HCOO*) with M=Al, Ga NCs at the PBE0-D3/def2TZVP level of theory (C/M = Charge/Multiplicity).

Al	COOH		Ga	COOH	
C/M	Isomer	Relative E (eV)	C/M	Isomer	Relative E (eV)
**2/2**	**S1**	**0.00**	**2/2**	**S1**	**0.00**
	S2	0.13		S2	0.43
	M	1.63		M	1.72
2/4	S1	0.09	2/4	S1	0.48
	S2	0.07		S2	0.43
	M	1.49			
	HCOO			HCOO	
2/2	S	0.53	2/2	S	1.36
	M	0.67		M	2.14

Table S38. Relative energies (eV) at different spin multiplicities and symmetry isomers (Conf) for the first intermediate of CO_2 reduction (*COOH or HCOO*) with M=In, Cr NCs at the PBE0-D3/def2TZVP level of theory (C/M = Charge/Multiplicity).

In	COOH		Cr	COOH	
C/M	Isomer	Relative E (eV)	C/M	Isomer	Relative E (eV)
**2/2**	**S1**	**0.00**	2/5	S1	0.13
	S2	0.24		S2	0.18
	M	1.64		M	1.30
2/4	S1	0.08	**2/7**	**S1**	**0.00**
	S2	0.13		S2	0.05
				M	1.41
	HCOO			HCOO	
2/2	S	1.25	2/7	S1	1.14
	M	1.30		M	1.74
	Br	2.02			

Reaction S2. $[Co_5MS_8(PEt_3)_5]^{n+} + CO_2 + 0.5H_2 \rightarrow [Co_5MS_8(PEt_3)_5COOH]^{n+}$ (*COOH); n = 1 or 2

Table S39. Reaction energies for COOH adsorption following reaction S2, n=1 (C/M = Charge/multiplicity, ZPE = Zero-point energy).

	C/M – L5	C/M - *COOH	Isomer	ΔE (eV)	ΔE_{ZPE} (eV)	$\Delta G_{298.15}$ (eV)
Co	1/4	1/3	S	-0.58	-0.34	0.35
Cr	1/7	1/6	S1	-0.42	-0.17	0.53
Mn	1/6	1/5	S	-0.60	-0.35	0.26
Fe	1/5	1/6	S	-0.50	-0.25	0.40
Ni	1/3	1/4	S	0.01	0.20	0.95
Cu	1/2	1/1	S	-0.44	-0.17	0.54
Mo	1/3	1/2	S1	-0.17	0.01	0.66
Pd	1/3	1/4	S	0.04	0.24	0.88
Al	1/2	1/1	S	-1.05	-0.76	-0.09
Ga	1/2	1/1	S	-0.85	-0.58	0.12
In	1/2	1/1	S	-0.63	-0.38	0.34

Reaction S3. *COOH + $0.5H_2 \rightarrow [Co_5MS_8(PEt_3)_5CO]^{n+}$ (*CO) + H_2O; n = 1 or 2

Reaction S4. *CO $\rightarrow [Co_5MS_8(PEt_3)_5]^{n+}$ + CO; n = 1 or 2

Table S40. Relative energies (eV) at different spin multiplicities for the second intermediate of CO_2 reduction (*CO) with M=Co, Fe, Cu, Al NCs at the PBE0-D3/def2TZVP level of theory (C/M = Charge/Multiplicity).

Co C/M	Relative E (eV)	Fe C/M	Relative E (eV)
1/2	0.02	1/3	0.77
1/4	**0.00**	1/5	**0.00**
1/6	0.45	1/7	0.06
**Cu C/M**	**Relative Energy (eV)**	**Al C/M**	**Relative Energy (eV)**
1/2	**0.00**	2/1	**0.00**
1/4	0.38	2/3	0.43
1/6	SCF	2/5	0.76

Table S41. Reaction energies for CO adsorption following reaction S3, n=1 (C/M = Charge/multiplicity, ZPE = Zero-point energy).

	C/M – L5	C/M - *CO	Isomer	ΔE (eV)	ΔE_{ZPE} (eV)
Co	1/4	1/4	1.98	1.95	1.55
Cr	1/7	1/7	1.98	1.95	1.60
Mn	1/6	1/6	2.00	1.98	1.73
Fe	1/5	1/5	2.00	1.97	1.60
Ni	1/3	1/3	2.14	2.14	1.78
Cu	1/2	1/2	1.86	1.81	1.44
Mo	1/3	1/3	1.97	1.92	1.64
Pd	1/3	1/3	2.14	2.13	1.79
Al	1/2	1/2	2.09	2.07	1.75
Ga	1/2	1/2	1.91	1.89	1.51
In	1/2	1/2	1.86	1.84	1.52

Table S42. Reaction energies for the release of CO(g) following reaction S4, n=1 (C/M = Charge/multiplicity, ZPE = Zero-point energy).

	C/M – L5	C/M - *CO	Isomer	ΔE (eV)	ΔE_{ZPE} (eV)
Co	1/4	1/4	-0.68	-0.72	-1.13
Cr	1/7	1/7	-0.84	-0.88	-1.35
Mn	1/6	1/6	-0.68	-0.74	-1.21
Fe	1/5	1/5	-0.78	-0.82	-1.23
Ni	1/3	1/3	-1.42	-1.45	-1.96
Cu	1/2	1/2	-0.69	-0.74	-1.21
Mo	1/3	1/3	-1.07	-1.04	-1.52
Pd	1/3	1/3	-1.46	-1.48	-1.89
Al	1/2	1/2	-0.32	-0.41	-0.89
Ga	1/2	1/2	-0.34	-0.42	-0.85
In	1/2	1/2	-0.50	-0.57	-1.09

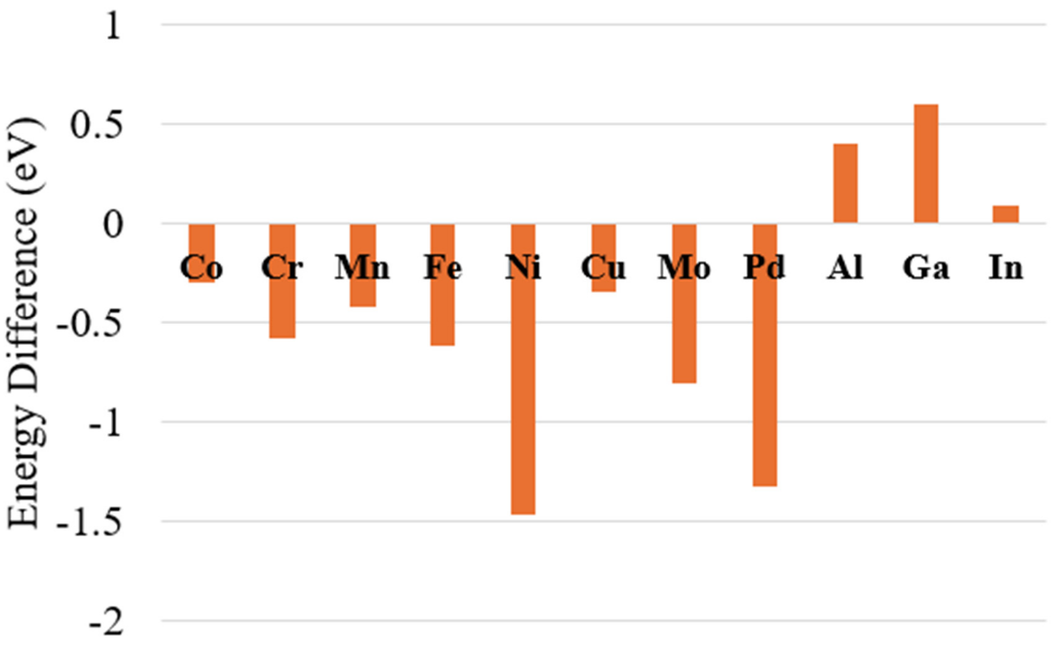

Figure S12. Difference in reaction energy of *COOH vs. *H formation on the S active sites for $[Co_5MS8(PEt3)5]1+$ NCs at the PBE0-D3/def2TZVP(opt)/CEP-31G(freq) level of theory. Positive values reflect selectivity towards CO (g) formation while negative values reflect selectivity towards H_2 (g) formation.

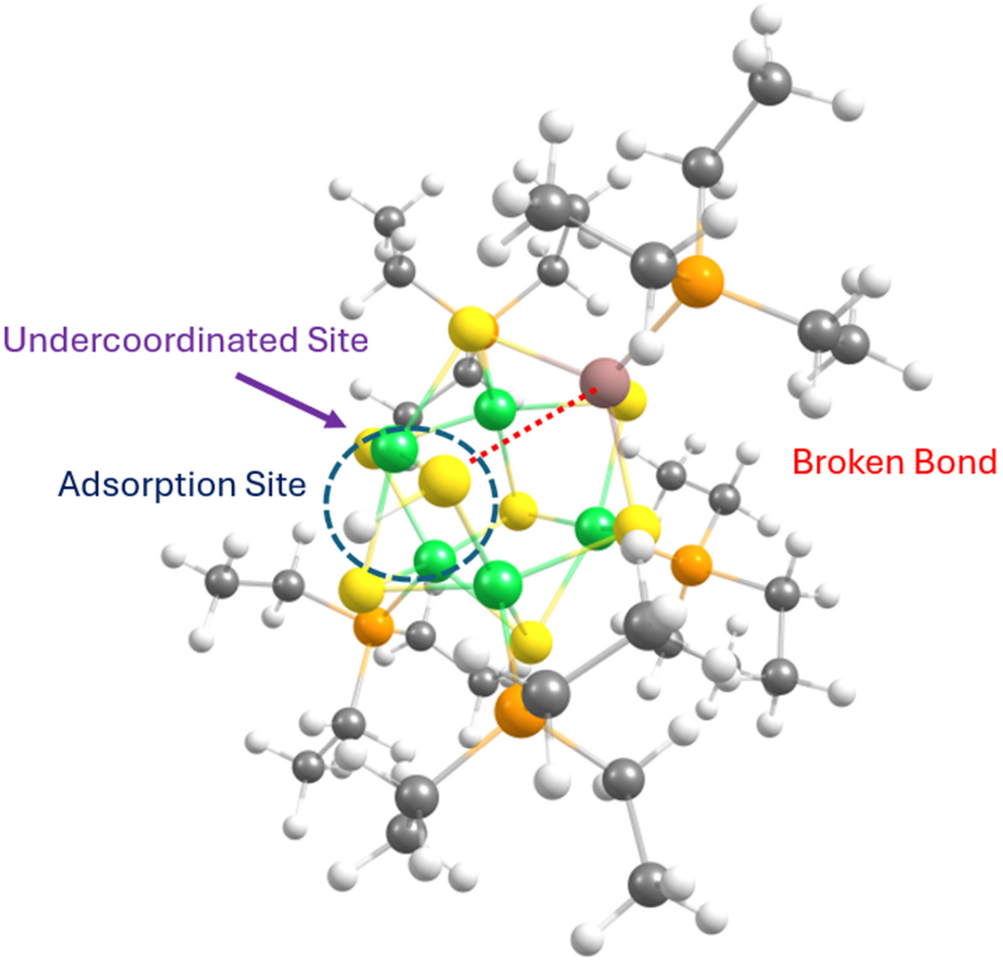

Figure S13. Pictorial image of bonding environment upon small molecule adsorption in $[Co_5MS_8(PEt_3)_5]^{2+}$ NCs.

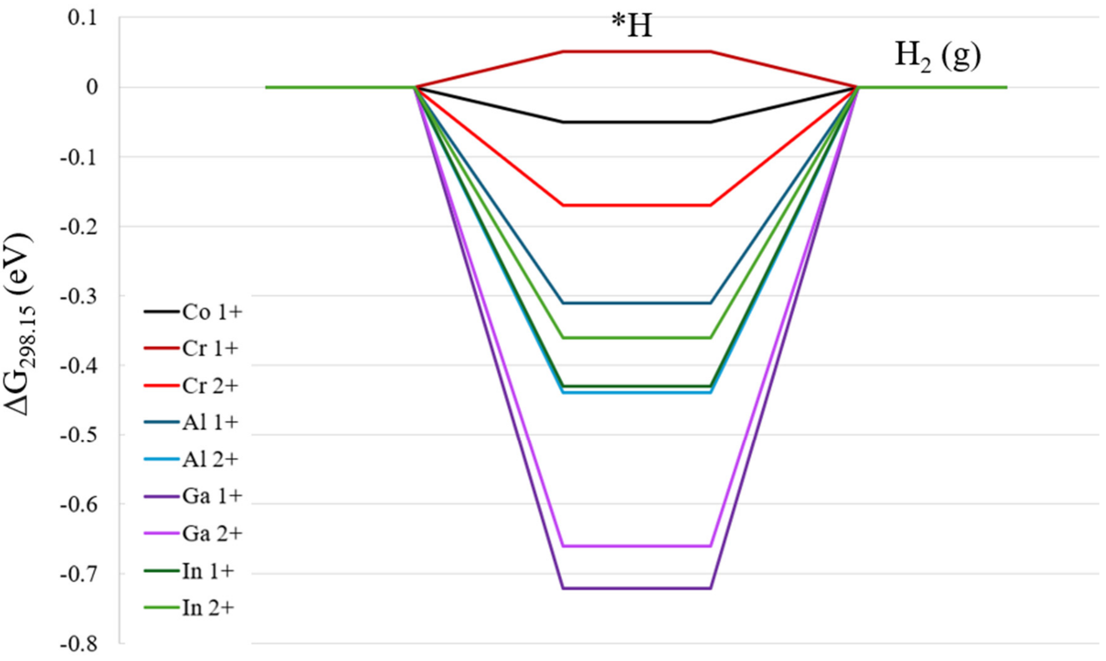

Figure S14. Reaction energy diagram of HER for $[Co_5MS_8(PEt_3)_5]^{2+}$ NCs at the PBE0-D3/def2TZVP(opt)/CEP-31G(freq) level of theory.

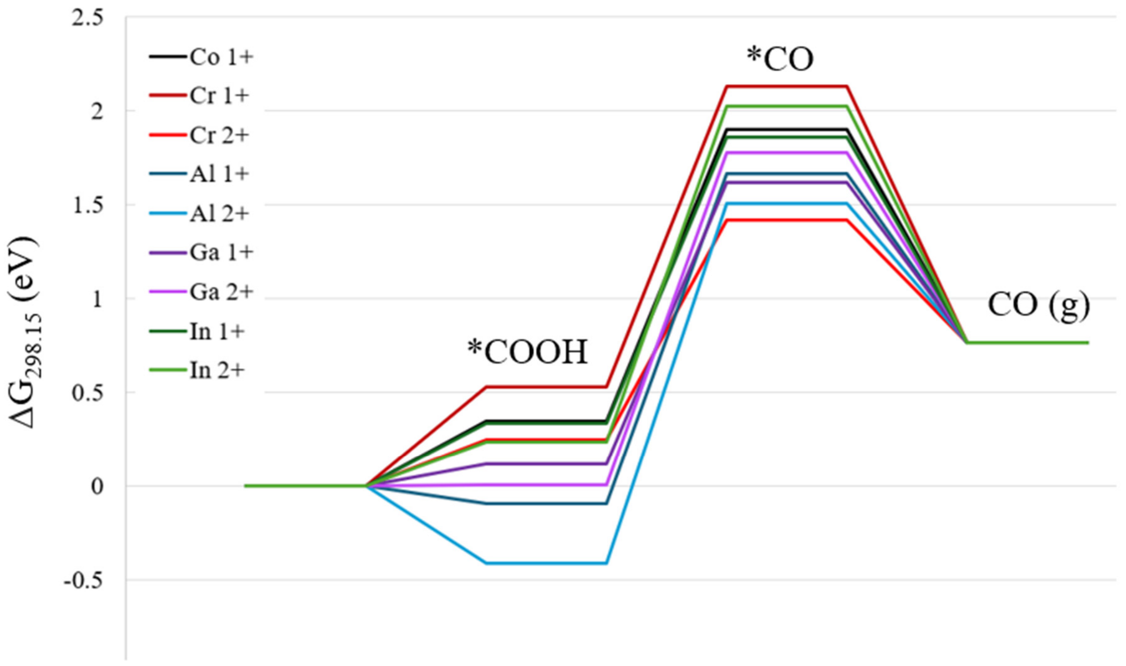

Figure S15. Reaction energy diagram of CO_2 reduction for $[Co_5MS_8(PEt_3)_5]^{2+}$ NCs at the PBE0-D3/def2TZVP(opt)/CEP-31G(freq) level of theory.

Reduction Potential (L_5) Nanoclusters:

Reaction S5. $[Co_5MS_8(PEt_3)_5]^+$ + e^- → $[Co_5MS_8(PEt_3)_5]^0$

Table S43. Energy calculation for $[Co_5MS_8(PEt_3)_5]^0$ (M=Co, Fe, Mn, Al) NCs at the PBE0-D3/def2TZVP level of theory (C/M = Charge/Multiplicity).

Co	Total Energy (ORCA)	**-14374.40**
C/M 0/3	ZPE Correction (Gaussian)	1.055834
	G Correction (Gaussian)	0.967169
	EZPE	-14373.34
	G	-14373.43
Fe	Total Energy (ORCA)	**-14255.35**
C/M 0/4	ZPE Correction (Gaussian)	1.056019
	G Correction (Gaussian)	0.965495
	EZPE	-14254.29
	G	-14254.38
Mn	Total Energy (ORCA)	**-14142.68**
C/M 0/5	ZPE Correction (Gaussian)	1.055275
	G Correction (Gaussian)	0.965062
	EZPE	-14141.62
	G	-14141.71
Al	Total Energy (ORCA)	**-13234.21**
C/M 0/1	ZPE Correction (Gaussian)	1.057332
	G Correction (Gaussian)	0.967471
	EZPE	-13233.16
	G	-13233.25
